# Weighted pedigree-based statistics for testing the association of rare variants

**DOI:** 10.1186/1471-2164-13-667

**Published:** 2012-11-24

**Authors:** Yin Yao Shugart, Yun Zhu, Wei Guo, Momiao Xiong

**Affiliations:** 1 Unit of Statistical Genomics, Division of Intramural Division Program, National Institute of Mental Health, National Institute of Health, Bethesda, MD, USA; 2 Division of Biostatistics, School of Public Health, The University of Texas Health Science Center at Houston, Houston, TX, USA; 3 Human Genetics Center, The University of Texas Health Science Center at Houston, P.O. Box 20186, Houston, TX 77225, USA

**Keywords:** Pedigree, Next-generation sequencing, GWAS, Rare Variants, Collapsing

## Abstract

**Background:**

With the advent of next-generation sequencing (NGS) technologies, researchers are now generating a deluge of data on high dimensional genomic variations, whose analysis is likely to reveal rare variants involved in the complex etiology of disease. Standing in the way of such discoveries, however, is the fact that statistics for rare variants are currently designed for use with population-based data. In this paper, we introduce a pedigree-based statistic specifically designed to test for rare variants in family-based data. The additional power of pedigree-based statistics stems from the fact that while rare variants related to diseases or traits of interest occur only infrequently in populations, in families with multiple affected individuals, such variants are enriched. Note that while the proposed statistic can be applied with and without statistical weighting, our simulations show that its power increases when weighting (WSS and VT) are applied.

**Results:**

Our working hypothesis was that, since rare variants are concentrated in families with multiple affected individuals, pedigree-based statistics should detect rare variants more powerfully than population-based statistics. To evaluate how well our new pedigree-based statistics perform in association studies, we develop a general framework for sequence-based association studies capable of handling data from pedigrees of various types and also from unrelated individuals. In short, we developed a procedure for transforming population-based statistics into tests for family-based associations. Furthermore, we modify two existing tests, the weighted sum-square test and the variable-threshold test, and apply both to our family-based collapsing methods. We demonstrate that the new family-based tests are more powerful than corresponding population-based test and they generate a reasonable type I error rate.

To demonstrate feasibility, we apply the newly developed tests to a pedigree-based GWAS data set from the Framingham Heart Study (FHS). FHS-GWAS data contain approximately 5000 uncommon variants with frequencies less than 0.05. Potential association findings in these data demonstrate the feasibility of the software PB-STAR (note, PB-STAR is now freely available to the public).

**Conclusion:**

Our tests show that when analyzing for rare variants, a pedigree-based design is more powerful than a population-based case–control design. We further demonstrate that a pedigree-based statistic’s power to detect rare variants increases in direct relation to the proportion of affected individuals within the pedigree.

## Background

In the last few years, researchers have conducted many Genome-Wide Association Studies (GWAS) to identify common variants underlying common human disorders. Although earlier analyses of GWAS data revealed that this approach can detect common variants with modest effects, only a small portion of significantly associated common variants prove to be functional. In addition, GWAS typically requires large sample sizes to achieve reasonable power [1].

Therefore, to detect rare variants associated with common disorders, researchers are increasingly turning to next generation sequencing (NGS) [2]. In recent years, advances in NGS technology have generated large amounts of data on the exome and on whole-genome sequencing, moving us ever closer to an understanding of how rare variants contribute to human traits and diseases. While NGS technology holds great promise, its platforms suffer from a number of drawbacks including high rates of calling error (particularly for the rare variants) and many missing values (due either to variants’ low quality or their location in difficult regions). However, the family-based designs proposed in this study, can be used to reduce error rates by detecting Mendelian errors and to impute missing values.

Statistical approaches currently available for the analysis of rare variants’ contributions to the development of complex traits include: the Combined Multivariate and Collapsing (CMC) Method [3], the Multivariate test of collapsed sub-groups, the Hotelling T^2^ test [4], MANOVA, the Fisher’s product method, the Weighted Sum-square (WSS) [5], the Kernel-Based Adaptive Test (KBAT) [6], the Variable-Threshold (VT) test [7]; the Sequence Kernel Association Test (SKAT) [8]; and the Functional Principal Component Test [9]. In addition, Neale et al. [10] proposed a method for testing the variance of the effects and Wu et al. [8] suggested a similar test using a slightly different approach. Han and Pan [11] modified Liu and Leal’s [3] original burden test to include the effect’s direction. More recently, Lin and Tang [12] have developed a generalized framework for the conduct of the statistical tests listed above. Researchers seeking to use different statistical methods to analyze NGS data may also wish to consult the following reviews of current methods for collapsing and pooling data: Bansal et al. [13], Basu and Pan [14], Feng et al. [15], and Lin and Tang [12].

Inasmuch as many common diseases such as cancer, cardiovascular disease, diabetes, immune disorders, and psychiatric disorders are known to cluster in pedigrees, there is a clear need to develop efficient statistical methods for analyzing sequence-based pedigree data. Yet despite its obvious importance, the use of pedigree-based collapsing methods to detect associations between diseases and rare variants in NGS-generated data has yet to be investigated in depth.

With the aim of finding how multiple rare variants within a genomic region contribute individually and collectively to disease, this study shows how collapsing techniques currently used to analyze population-based data can be adapted for the analysis of pedigree-based data. In our study design, therefore, all rare variants within a gene or a genomic region in pedigree data or a combination of pedigree and case–control data are collapsed into an overall variable.

To accomplish this aim, we developed a new pedigree-based method of association analysis for rare variants. Following the work of Thornton and McPeek [16], which used case–control association tests of common variants in related individuals**,** we devised a novel weighted statistic to compare affected and unaffected individuals within pedigrees using the value of their integrated overall variables, weighted by their *Identity by Descent* (IBD) coefficients. To evaluate the performance of this new method, we use simulations with varied pedigree structures to compute the type I error rates and power under different disease models. Our simulation results demonstrate that the proposed new method can be used with data from various study designs including case–control, sib-pairs, nuclear families, and multi-generation families.

This manuscript introduces several new methods for the statistical analysis of pedigree-based data. These include new ways to estimate allele frequency and a kinship matrix from genotype data, statistics for collapsing family-based data, and a correction factor for relatedness affected and unaffected pairs within pedigrees. Using simulations with seven types of data structures, we evaluate our test statistics for impact of sample size, proportion of risk variants, and proportion of variants with effects in opposite directions, on type I error rates, and analytical power for detecting rare-variant association. After these evaluation tests and demonstrations, we conclude with a summary of our statistics’ merits and limitations.

## Methods

For our readers’ convenience, we have included a glossary for parameters and definitions used in equations in Table 1.

**Table 1:** Glossary of parameters

**Notations**	**Meaning**
subscript	Individuals
i, j = 1,…,n	
subscript k = 1,…,m	variant/marker
s	Iteration
*p*_*k*_	frequency of the reference allele of the k-th variant
*x*_*ik*_ = 0,1,2	indicator variable of genotype for the *k-th* variant of the *i-th* individual
Φ	kinship matrix
superscript T	matrix transpose
*z*_*i*_	indicator variable of presence of rare variants in the region for the *i*-th individual
*h*_*i*_	inbreeding coefficient of individual *i*
*γ*_2*k*_, *γ*_1*k*_	relative risks
*P*_*corr*_	correction factor in the test statistics accounting for the relatedness
*n*_*G*_	number of controls
*n*_*c*_	number of cases
*p*	Pr(presence of rare variants in the genomic region)
*T*_*C*_	population-based collapsing test statistic
*T*_*CF*_	family-based collapsing test statistic
*T*_*WSS*_	population-based weighted sum statistic
*T*_*WSSF*_	family-based weighted sum statistic
*T*_*VT*_	population-based variant threshold statistic
*T*_*VTF*_	family-based variant threshold statistic

### Estimation of kinship matrix when allele frequencies are known

Consider *m* markers. Let *x*_*ik*_ be the indicator variable of genotype for the *k-th* variant of the *i-th* individual, and the values are taken to be 0, 1 and 2 as the number of reference alleles. Let *p*_*k*_ be the frequency of the reference allele of the *k*-th variant (the allele frequency is the count of reference allele over the sum of two alleles in all individuals at a particular marker). The kinship coefficient matrix (Φ) is given by

$$\Phi =\left[\begin{array}{cccc} \varphi_{11} & \varphi_{12} & \cdots & \varphi_{1n}\\ \varphi_{21} & \varphi_{22} & \cdots & \varphi_{2n}\\ \cdots & \cdots & \cdots & \cdots \\ \varphi_{n1} & \varphi_{n2} & \cdots & \varphi_{\mathrm{nn}} \end{array}\right]\;\text{,}$$

where *ϕ*_*ij*_ is the kinship coefficient between individual *i* and *j* In cases where the kinship matrix Φ quantifying relatedness among individuals is unknown, it can be estimated from genetic variants in the data. Recently, Yang et al. [17] derived equations to estimate the genealogy matrix (defined as genetic relationship matrix between pairs of individuals which mathematically equals 2Φ_)._ We simply followed the equation in Yang et al. [17] as:

(1a) $$\begin{array}{l} \psi_{\mathrm{ij}}=\frac{1}{m}\sum_{k=1}^{m}\frac{\left(x_{\mathrm{ik}}-2p_{k}\right)\left(x_{\mathrm{jk}}-2p_{k}\right)}{2p_{k}\left(1-p_{k}\right)},i\neq j\\ \psi_{\mathrm{ii}}=1+\frac{1}{m}\sum_{k=1}^{m}\frac{x_{\mathrm{ik}}^{2}-\left(1+2p_{k}\right)x_{\mathrm{ik}}+2p_{k}^{2}}{2p_{k}\left(1-p_{k}\right)},i=j\text{.} \end{array}$$

The kinship coefficients are estimated by

(1b) $$\phi_{\mathrm{ij}}=\frac{1}{2}\psi_{\mathrm{ij}}\text{.}$$

In the presence of inbreeding, the estimated *ψ*_*ii*_ is greater than 1 (in the manuscript by Yang et al., this is refer to as the “background effect”).

### Estimation of kinship matrix when the population allele frequencies are not known

When estimates of allele frequencies based on population data are not available (i.e. variants that have not been genotyped in reference datasets such as 1000 Genomes or HapMap), we estimate the allele frequencies using the genetic marker information from pedigree members. An iterative algorithm initialized with the observed frequency across pedigrees is used to estimate these frequencies. We note that the use of rare variants could lead to unstable estimates of kinship coefficients, therefore, only common variants should be used for the estimation.

Step 1 (Initialization): Use the allele frequency computed in all pedigree members as ${\hat{p}}_{k}$ to estimate the kinship matrix Φ_(0)_.

Step 2 (Iteration) Let k be the *k*-th variant in the genomic region. For the *s*-th iteration, we conduct the following steps:

a) Use Φ_(s)_ to estimate ${\hat{p}}^{\left(s\right)}$, ${\hat{p}}_{k}^{\left(s\right)}={\left(1^{T}{\Phi_{\left(s\right)}}^{-1}1\right)}^{-1}1^{T}{\Phi_{\left(s\right)}}^{-1}{\left(x_{1k,}x_{2k,\ldots ,}x_{\mathrm{nk}}\right)}^{T}$ where **1** is a vector of **1**’s and (*x*_1*k*_*, x*_2*k*_*,…,x*_*nk*_) is a vector of the indicator variable for genotypes at the *k*-th variant in the genomic region as defined above (*k* = 1,…,*m*).

b) Use this ${\hat{p}}^{\left(s\right)}$ to estimate Φ_(s+1)_.

c) Stop at convergence or at a predetermined maximum iteration limit.

### Collapsing method fundamentals

We extend the population-based collapsing test to families with either known or unknown population structures. Let *n* be the number of individuals in the sampled pedigrees, an indicator variable for the *i*-th individual in the pedigrees is defined as

$$z_{i}=\{\begin{array}{ll} 1 & \text{if rare variants are present in the region}\\ 0 & \text{otherwise} \end{array}\text{,}$$

where *i* = 1, …, *n*.

Let *Z* = [z_1_, z_2_,…, *z*_*n*_]^*T*^. Under the null hypothesis (the genomic region has no association with the disease), the expectation of the vector of indicator variables is given by:

$$E_{0}\left[Z\right]={\left[p,p,\ldots ,p\right]}^{T}\text{,}$$

where *p* = Pr(presence of rare variants in the genomic region). If we reject the null hypothesis, it is assumed that

$$E\left[z_{i}\right]=\mu_{i}=p+u_{i}r\text{,}$$

where

$$\begin{array}{l} 0<p<1,0<p+r<1,\text{and}\\ \;u_{i}=\left\{\begin{array}{ll} 1 & \text{if the}\;i^{\mathrm{th}}\;\text{individual is affected}\\ 0 & \text{otherwise.} \end{array}\right) \end{array}$$

We define *μ* = [*μ*_1_, *μ*_2_, …, *μ*_*n*_]^*T*^. The partial derivative of *μ* with respect to *p* is given by

$$D_{p}=\frac{\partial \mu }{\partial p}={\left[1,1,\ldots ,1\right]}^{T}\text{.}$$

Similarly, we have $D_{r}=\frac{\partial \mu }{\partial r}=u\text{,}$ where *u =* [*u*_1_*, u*_2_*, …, u*_*n*_]^*T*^.

Next, we calculate the covariance matrix of the vector Z. Let *h*_*i*_ be the inbreeding coefficient of individual *i*. Let *σ*^2^ = *p*(1–*p*). For computing the expectations by conditioning, we have

(2a) $$\begin{array}{c} \;Cov(z_{i},z_{j})=E\left[z_{i}z_{j}\right]-E\left[z_{i}\right]E\left[z_{j}\right]\\ \;=E\left[E\left[z_{i}z_{j}|z_{i}\right]\right]-E\left[z_{i}\right]E\left[E\left[z_{j}|z_{i}\right]\right]\\ \;=\varphi_{\mathrm{ij}}E\left[z_{i}^{2}\right]-\varphi_{\mathrm{ij}}{\left(E\left[z_{i}\right]\right)}^{2}\\ =\varphi_{\mathrm{ij}}\sigma^{2}\text{.} \end{array}$$

By the same token, we have

(2b) $$\text{Var}\;\left(z_{i}\right)=\left(1+h_{i}\right)\sigma^{2}=\varphi_{\mathrm{ii}}\sigma^{2}\text{,}$$

The kinship coefficients in equations (2a) and (2b) are estimated by equation (1a) and (1b), where the inbreeding coefficient *h*_*i*_ of individual i can be estimated by *ϕ*_*ii*_–1.

Combining equations (2a) and (2b), we can obtain the following covariance matrix of vector Z:

(3) $$\Sigma =\text{Var}\left(Z,Z\right)=\sigma^{2}\Phi \text{.}$$

Let

$$H_{C}={\left(D_{r}-\frac{n_{c}}{n}D_{p}\right)}^{T}Z\text{,}$$

where *n*_*c*_ is the number of cases and the variance of *H*_*C*_ is given by

$$\begin{array}{l} \Gamma =\text{Var}\;\left(H_{C},H_{C}\right)\\ \;={\left(D_{r}-\frac{n_{c}}{n}D_{p}\right)}^{T}\Phi \left(D_{r}-\frac{n_{c}}{n}D_{p}\right)\sigma^{2}\text{.} \end{array}$$

The statistic for testing the association of a genomic region containing the disease locus can be defined as

(4) $$T_{\mathrm{CF}}=\frac{H_{C}^{2}}{\Gamma }\text{.}$$

However,

(5) $$\begin{array}{l} H_{C}=D_{r}^{T}Z-\frac{n_{c}}{n}D_{p}^{T}Z\\ \;=\sum_{i\in \text{cases}}z_{i}-\frac{n_{c}}{n}\sum_{i=1}^{n}z_{i}\\ \;=n_{c}{\bar{Z}}_{A}-\frac{n_{c}}{n}\left(n_{c}{\bar{Z}}_{A}+n_{G}{\bar{Z}}_{G}\right)\\ \;=\frac{n_{c}n_{G}}{n}\left({\bar{Z}}_{A}-{\bar{Z}}_{G}\right)\text{,} \end{array}$$

where *n*_*G*_ is the number of controls, ${\bar{Z}}_{A}{\;\text{and}\;\bar{Z}}_{G}$ are the averages of indicator variables in cases and controls, respectively. The test statistic can then be rewritten as:

(6) $$\begin{array}{l} T_{\mathrm{CF}}=\frac{\frac{n_{c}n_{G}}{n}\frac{{\left({\bar{Z}}_{A}-{\bar{Z}}_{G}\right)}^{2}}{\sigma^{2}}}{\frac{n}{n_{c}n_{G}}{\left(D_{r}-\frac{n_{c}}{n}D_{p}\right)}^{T}\Phi \left(D_{r}-\frac{n_{c}}{n}D_{p}\right)}\\ \;=\frac{T_{C}}{P_{\mathrm{corr}}}\text{,} \end{array}$$

where *T*_*C*_ is the population-based collapsing test statistic and $P_{\mathrm{corr}}=\frac{n}{n_{c}n_{G}}{\left(D_{r}-\frac{n_{c}}{n}D_{p}\right)}^{T}\Phi \left(D_{r}-\frac{n_{c}}{n}D_{p}\right)$ is a correction factor. Under the null hypothesis of no association, *T*_*CF*_ is distributed as a central *χ*_(1)_^2^ distribution. It follows that when the correction factors are computed using the IBD information, the relatedness effect (if present) can be easily corrected.

Similarly, population-based weighted sum (WSS) and variant threshold (VT) tests can also be extended to pedigrees:

$$T_{\mathrm{WSSF}}=\frac{T_{\mathrm{WSS}}}{P_{\mathrm{corr}}}\;\text{and}\;\frac{T_{\mathrm{VT}}}{P_{\mathrm{corr}}}\text{.}$$

### Single marker analysis

Although the main focus of this investigation is to develop weight-based collapsing statistics to analyze for rare variants in families, for comparison, we also use a Chi-squared test to calculate an individual p-value for each variant in a given gene. For every gene considered, we select the variant with the lowest p-value and then permute the disease-normal status 5000 times to obtain an empirical p value for the selected variant. This permutation test is conducted using the following mathematical formula.

Let *P*_min_ be the minimum p value of the Chi-square tests among all variants in a gene. Let $p_{\text{mim}}^{\left(1\right)},\ldots ,{\text{pmim}}^{\left(5000\right)}$ be the minimum p value in 5000 permutations. The empirical p value can be expressed as $\sum_{b=1}^{5000}I\left(P_{\text{min}}^{\left(b\right)}\leq P_{\text{min}}\right)/5000$.

#### Using simulation to estimate power and type I error rate

In this study, the forward evolutionary simulation tool *ForSim*[18] was used to simulate genetic data taking pedigree structures and evolutionary processes (such as natural selection, mutation rate and population demographics) into account. These simulated data were then analyzed with our PB_STAR software to calculate the power and type I error rates for family-based single marker analysis (using a Chi-square test) and for two collapsing methods: WSS and VT. Under four simulation models (dominant, multiplicative, additive and recessive), the mutation rate was assumed to be 2.5 × 10^-8^. We set the total number of generations as 100, the recombination rate as 1 cM per Mb, the disease prevalence as 0.09 and the growth rate as 2.1. Parameters were set to simulate the desired pedigrees with a fixed ratio of affected and unaffected individuals within a pedigree.

*ForSim* is a flexible software package that allows users to re-define case or control status by making specific assumptions about disease frequency and penetrance when associated with dominant, recessive and multiplicative models. When we later re-assigned case status using a penetrance function, we found that, changing simulation parameters does not significantly impact either power or type I error rates (data not shown).

*ForSim* also allows generation of hundreds of functional variants in two unlinked genes, with only one gene relevant to the disease phenotype of interest. All variants were presumed to influence the disease in an additive fashion. Variants arising by mutation were assigned effect sizes. In this way, we simulated 100 generations of a single population, allowing variants to accumulate until the last generation, which showed a total disease prevalence of 0.09. From this set of pedigrees, we randomly sampled for six types of desired pedigree, each with at least two affected individuals. The procedure for calculating the type I error rate and power is detailed below.

### Type I error rate

To assess type I error rates of the test statistics, we simulated seven settings of data with different sample sizes and pedigree designs: 1) a population design with equal number of cases and controls (case–control design); 2) Sib-pair families without parental genotypes, ratio of affected/unaffected is 1 (Sib-pair-1); 3) sib-pair families without parental genotypes, ratio of affected/unaffected is 2 to 1 (Sib-pair-2); 4) nuclear families with offspring, ratio of affected/unaffected is 1 (Nuclear-family-1); 5) nuclear families with offspring, ratio of affected/unaffected is 2 (Nuclear-family-2); 6) three generation families with children and grandchildren, ratio of affected/unaffected is 1 (Three-generation-1) and 7) Three generation families with children and grandchildren, ratio of affected/unaffected is 2 (Three-generation-2). To calculate type I error rates, 5000 simulated replicates were performed for each design. “Rare variants” were defined as variants with Minor Allele Frequency (MAF) of less than 1%.

### Power

To evaluate the power of the proposed test statistics by simulation, we had first to determine disease status based upon individual genotype and penetrance at each locus. Each group’s population attributable risk (PAR) was set as 0.006 [19], the genotype relative risk was set to be inversely proportional to its MAF. It was further assumed that the baseline penetrance of the wild-type genotype is equal across all variants sites and that variants influence disease susceptibility independently (i.e. with no epistasis). More specifically, at the *k*-th variant site, let *γ*_2*k*_ be the relative risk for genotype 2, and let *γ*_1*k*_ be the relative risk for genotype1. For the dominant model: *γ*_2*k*_ = *γ*_1*k*_, for the additive model: *γ*_2*k*_ = 2*γ*_1*k*_–1, for the multiplicative model: *γ*_2*k*_ = *γ*_1*k*_^2^, and for the recessive model: *γ*_1*k*_ = 1. Seven design settings were simulated under these four different models. We assigned each individual to either a case or control groups depending upon their “disease status”. We also varied study design and pedigree structure in our simulations to see how sample size and proportion of causal variants (PCV) to non-causal variants (NCV) affect the power of test statistics and to provide practical guidelines for sampling.

### Weights

Madsen and Browning [5] proposed analyzing for rare variants using a collapsing method with weights based on variant frequency. Because these weights depend on phenotypic values, they further suggested a permutation-based test to calculate p-values. Although it also requires the use of permutation to calculate p-values, the VT method, by contrast, does not rely on assumptions about the distribution of effect size. In this study, both WSS and VT were used to analyze our simulated data and to calculate p-values based upon permutations. Obviously, more permutation runs are likely to lead to more precise estimation of power, although the computational burden is also increasingly greater. In this study, estimation of power is based upon 5000 permutation runs.

In addition evaluations based on results from the seven simulation designs described above, we used our test statistics in two additional simulations, whose mixed population designs more closely resemble those found in actual studies. The first design is a mix of 33% Sibpair-2 families, 33% Nuclear-2 families, and 34% Three-generation-2 families (Mix-1). The second design is a mix of 50% Sib-pair-2 families and 50% Nuclear-2-families (Mix-2). We compared the power of two mixed designs and un-mixed designs using simulation.

## Results

In this section, we present the results from tests assessing the power and type I error rate of our proposed method. The following section describes our tests for the effects of sample size, the proportion of risk variants, and variants functioning in opposite directions in seven different simulated pedigree settings.

### Empirical Type I error rates

To evaluate type I error rates, we consider two scenarios for relatedness of individuals. In the first scenario, we use theoretical kinship coefficients between pairs of individuals in the same pedigrees as our kinship coefficients, assuming that kinship coefficients between pairs of individuals who are in different pedigrees are zero. In the second scenario, whether or not paired individuals are from the same pedigree, all kinship coefficients between pairs of individuals are estimated by genotyped variants. These tests show that in both single-marker and collapsing tests, failure to correct for population structure results in inflated type I error rates. Simulation results also indicate that with or without weights, Type I error rates for all collapsing tests do not deviate from the nominal level (Table 2).

**Table 2:** Type I error rates

**Study Design**	**Nominal Level**	**Estimated Kinship Coefficient**	**Theoretic Kinship Coefficient**	**Without Correction for Relatedness**
Population Design with equal number of case and control	0.050	0.0515	0.0480	0.0505
	0.010	0.0096	0.0099	0.0099
	0.001	0.0010	0.0010	0.0010
Mixed family and case–control design	0.050	0.0504	0.0494	0.0620
	0.010	0.0102	0.0097	0.0160
	0.001	0.0010	0.0010	0.0015
Sib-pair-1	0.050	0.0486	0.0475	0.0813
	0.010	0.0097	0.0092	0.0129
	0.001	0.0010	0.0011	0.0012
Nuclear-family-1	0.050	0.0531	0.0497	0.0829
	0.010	0.0093	0.0093	0.0107
	0.001	0.0010	0.0009	0.0014
Three-generation-1	0.050	0.0512	0.0484	0.0874
	0.010	0.0094	0.0102	0.0099
	0.001	0.0010	0.0010	0.0019

5000 replicates were conducted to calculate type I error rates for each study design.

Calculations further show similar type I error rates regardless of pedigree structure (hybrid design, sib-pair, nuclear family, or three-generation family). Even after correction factors (calculated using estimated or true IBD coefficients) are applied, type I error rates do not differ significantly from nominal levels (α = 0.05, 0.01, and 0.001), regardless of the type of collapsing methods used. (See Table 2 for results from our type I error rate validity tests in a hybrid design (N = 2100), in which half the data come from nuclear families).

### Analytic power

To test the analytic power of our proposed method, we conducted three sets of simulations in which four statistics (corrected single-marker Chi-squares, family-based collapsing methods, VT, and WSS) are used to analyze for four disease models (dominant, additive, multiplicative, and recessive).

**Figure 1:**
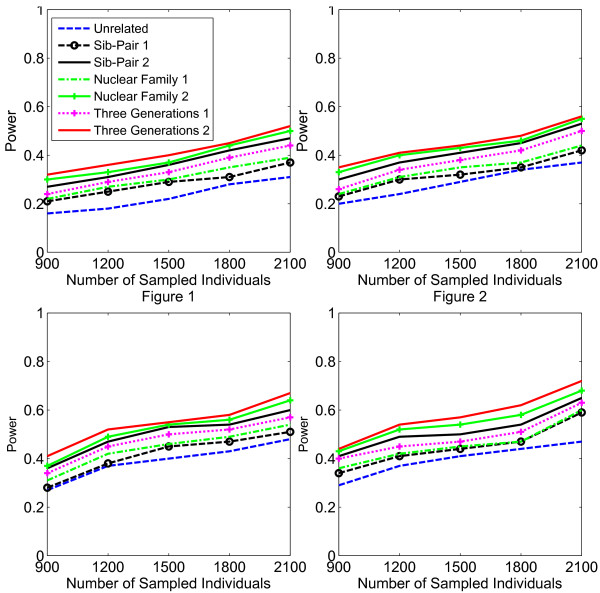
**The power curves of the family-based corrected single marker χ**^**2 **^**test statistic as a function of the total number of individuals at the significance level α = 0.05 in the test under seven settings: unrelated individuals in cases-controls study, nuclear family groups 1 and 2, sib-pair groups 1 and 2 and three generation family groups 1 and 2, assuming a dominant model, 20% of the risk variants and a baseline penetrance of 0.01.**

**Figure 2:**
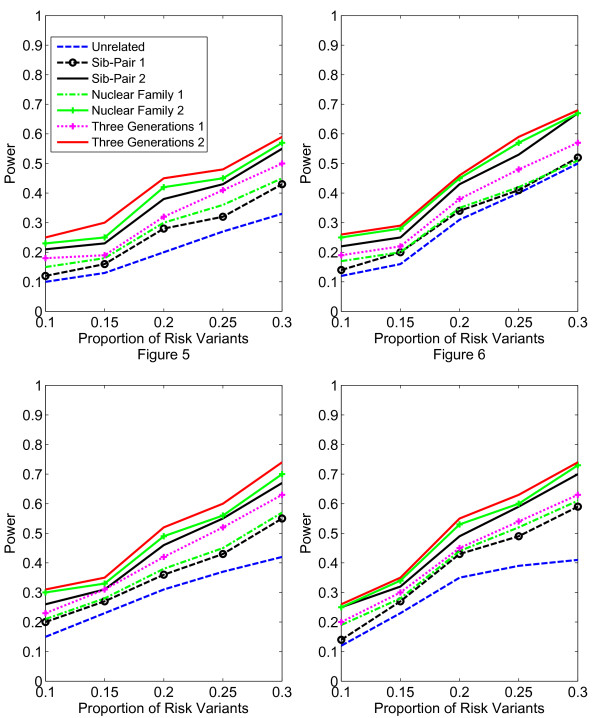
The power curves of the family-based collapsing test (variants with frequencies ≤0.005 were collapsed) statistic as a function of the total number of individuals at the significance level α = 0.05 in the test under seven settings: unrelated individuals in cases-controls study, nuclear family groups 1 and 2, sib-pair groups 1 and 2 and three generation family groups 1 and 2, assuming a dominant model, 20% of the risk variants and a baseline penetrance of 0.01.

**Figure 3:**
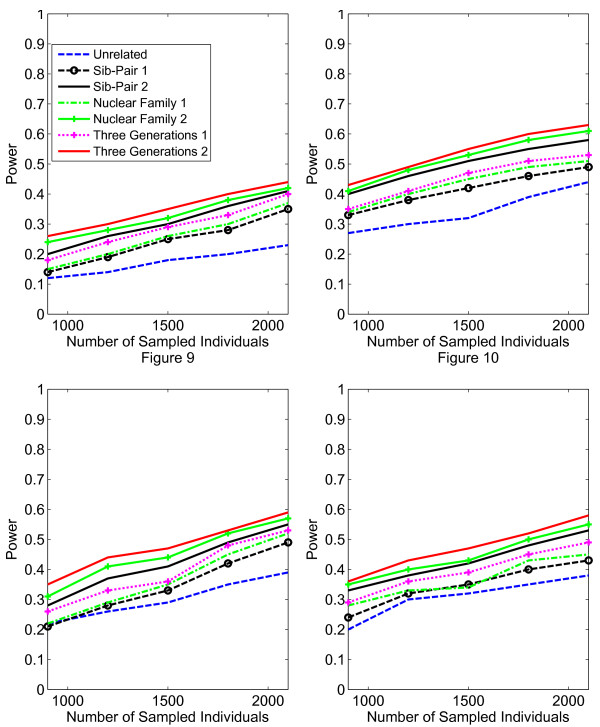
The power curves of the family-based VT test statistic as a function of the total number of individuals at the significance level α = 0.05 in the test under seven settings: unrelated individuals in cases-controls study, nuclear family groups 1 and 2, sib-pair groups 1 and 2 and three generation family groups 1 and 2, assuming a dominant model, 20% of the risk variants and a baseline penetrance of 0.01.

**Figure 4:**
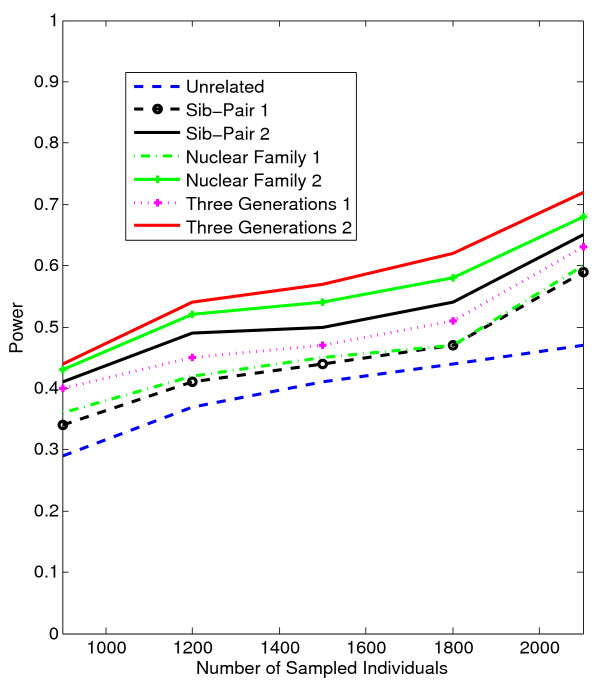
The power curves of the family-based WSS test statistic as a function of the total number of individuals at the significance level α = 0.05 in the test under seven settings: unrelated individuals in cases-controls study, nuclear family groups 1 and 2, sib-pair groups 1 and 2 and three generation family groups 1 and 2, assuming a dominant model, 20% of the risk variants and a baseline penetrance of 0.01.

**Figure 5:**
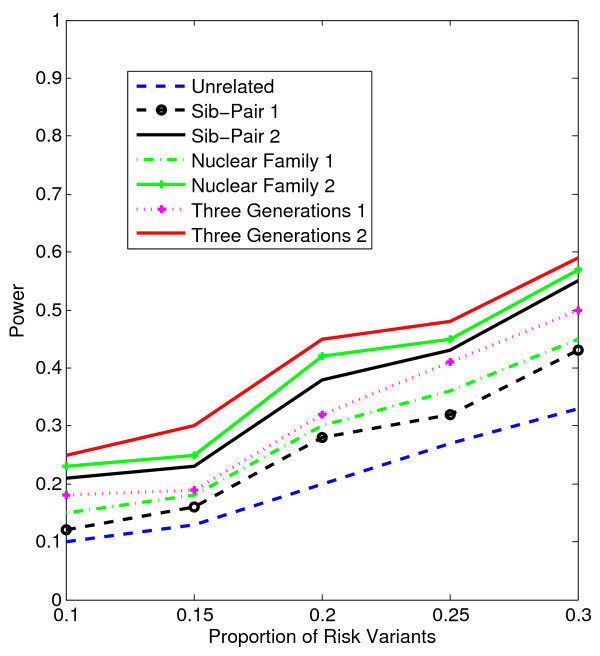
**The power curves of the family-based corrected single marker χ**^**2 **^**test statistic as a function of the proportion of risk variants at the significance level α = 0.05 in the test under seven settings: unrelated individuals in cases-controls study, nuclear family groups 1 and 2, sib-pair groups 1 and 2 and three generation family groups 1 and 2, assuming a dominant model, a total of 1,800 sampled individuals and a baseline penetrance of 0.01.**

**Figure 6:**
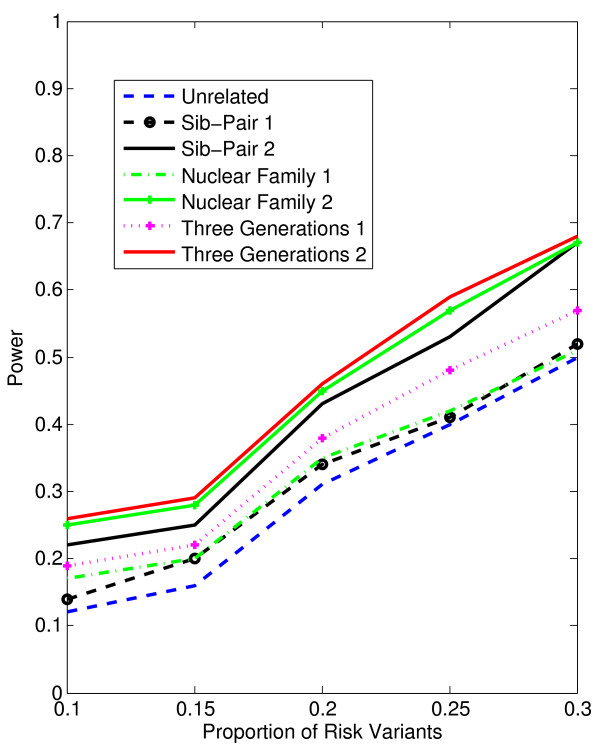
The power curves of the family-based collapsing test (variants with frequencies ≤0.005 were collapsed) statistic as a function of the proportion of risk variants at the significance level α = 0.05 in the test under seven settings: unrelated individuals in cases-controls study, nuclear family groups 1 and 2, sib-pair groups 1 and 2 and three generation family groups 1 and 2, assuming a dominant model, a total of 1,800 sampled individuals and a baseline penetrance of 0.01.

**Figure 7:**
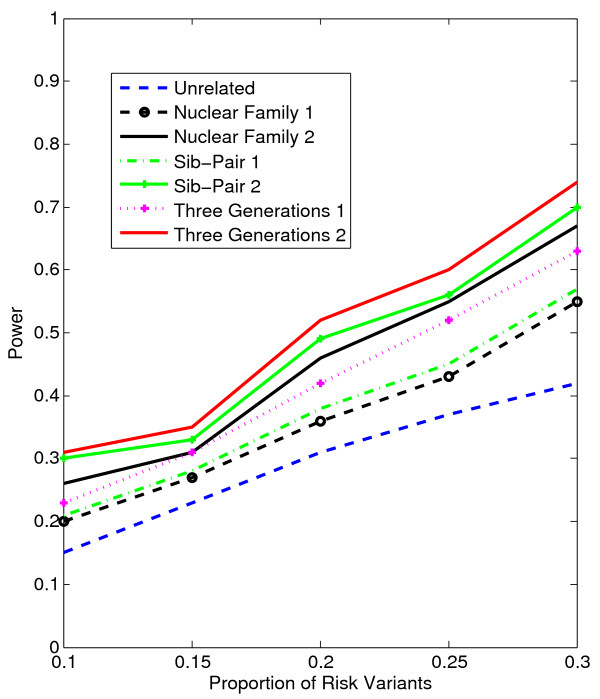
The power curves of the family-based VT test statistic as a function of the proportion of risk variants at the significance level α = 0.05 in the test under seven settings: unrelated individuals in cases-controls study, nuclear family groups 1 and 2, sib-pair groups 1 and 2 and three generation family groups 1 and 2, assuming a dominant model, a total of 1,800 sampled individuals and a baseline penetrance of 0.01.

**Figure 8:**
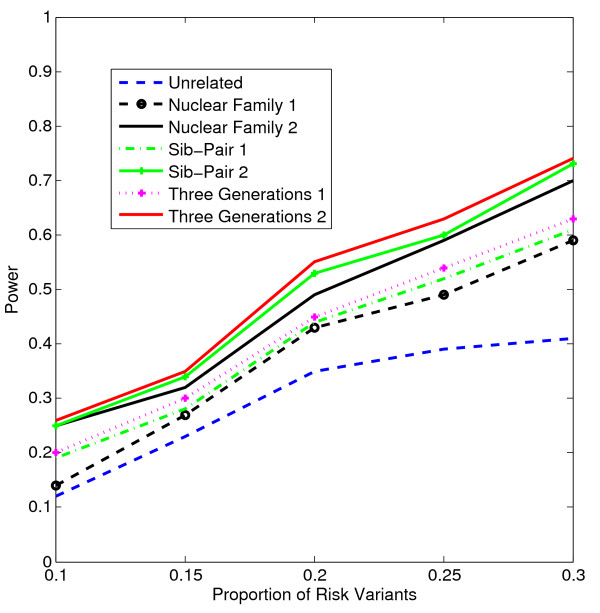
The power curves of the family-based WSS test statistic as a function of the proportion of risk variants at the significance level α = 0.05 in the test under seven settings: unrelated individuals in cases-controls study, nuclear family groups 1 and 2, sib-pair groups 1 and 2 and three generation family groups 1 and 2, assuming a dominant model, a total of 1,800 sampled individuals and a baseline penetrance of 0.01.

**Figure 9:**
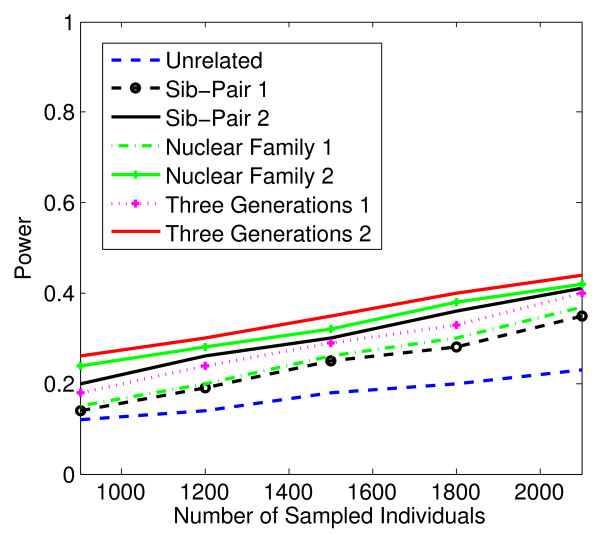
**The power curves of the family-based corrected single marker χ**^**2 **^**statistic under opposite directions of association as a function of the total number of individuals at the significance level α = 0.05 in the test under seven settings: unrelated individuals in cases-controls study, nuclear family groups 1 and 2, sib-pair groups 1 and 2 and three generation family groups 1 and 2, assuming a dominant model, 20% of the risk variants and a baseline penetrance of 0.01.**

**Figure 10:**
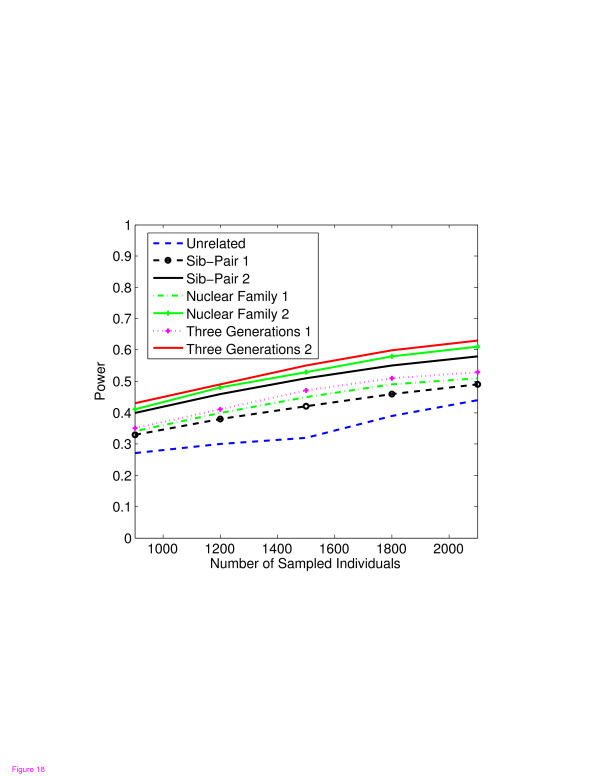
The power curves of the family-based collapsing test (variants with frequencies ≤0.005 were collapsed) statistic under opposite directions of association as a function of the total number of individuals at the significance level α = 0.05 in the test under seven settings: unrelated individuals in cases-controls study, nuclear family groups 1 and 2, sib-pair groups 1 and 2 and three generation family groups 1 and 2, assuming a dominant model, 20% of the risk variants and a baseline penetrance of 0.01.

**Figure 11:**
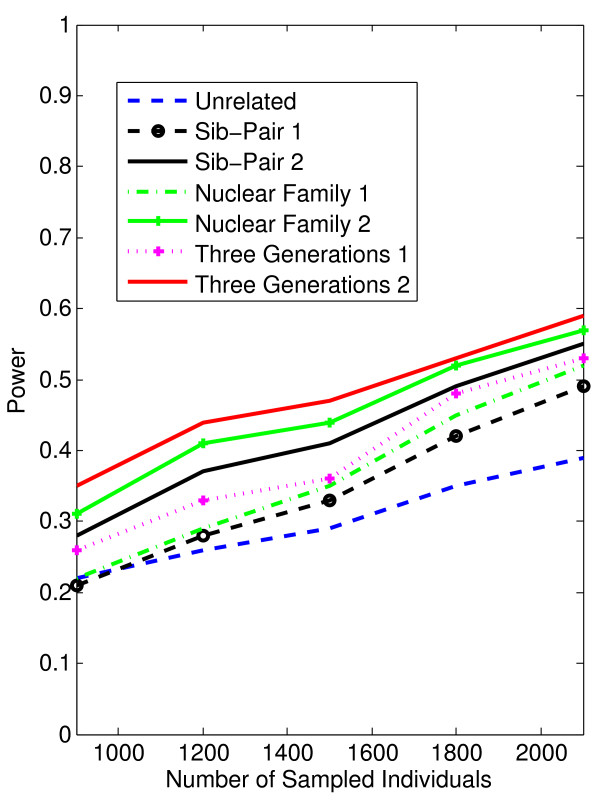
The power curves of the family-based VT statistic under opposite directions of association as a function of the total number of individuals at the significance level α = 0.05 in the test under seven settings: unrelated individuals in cases-controls study, nuclear family groups 1 and 2, sib-pair groups 1 and 2 and three generation family groups 1 and 2, assuming a dominant model, 20% of the risk variants and a baseline penetrance of 0.01.

**Figure 12:**
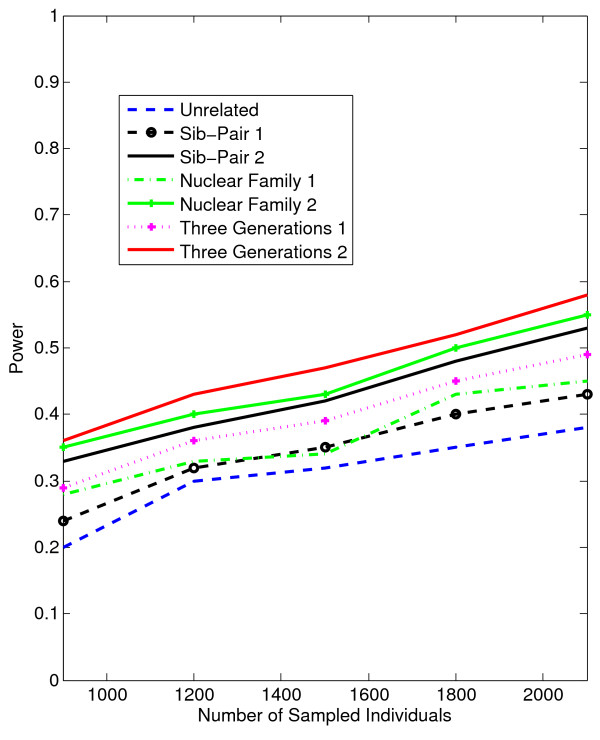
The power curves of the family-based WSS test statistic under opposite directions of association as a function of the total number of individuals at the significance level α = 0.05 in the test under seven settings: unrelated individuals in cases-controls study, nuclear family groups 1 and 2, sib-pair groups 1 and 2 and three generation family groups 1 and 2, assuming a dominant model, 20% of the risk variants and a baseline penetrance of 0.01.

In Figures 1, 2, 3, 4, the X axis stands for sample size, which varies from 900 to 2100. “1” indicates single marker test; “2” indicates family-based collapsing test; “3” indicates family-based VT test; “4” indicates family-based WSS test. In Figures 5, 6, 7, 8, the X axis stands for the proportion of risk variants. “5” indicates single marker test; “6” indicates family-based collapsing test; “7” indicates family-based VT test; “8” indicates family-based WSS test. In Figures 9, 10, 11, 12, the X axis stands for the sample size when the variants with effect of opposite side are considered. “9” indicates single marker test; “10” indicates family-based collapsing test; “11” indicates family-based VT test; “12” indicates family-based WSS test)In all instances, total trend significance level of alpha = 0.05. To reduce the number of graphs presented in the main body of this manuscript, power calculations for additive, multiplicative, and recessive models appear as Additional files 1, 2, 3, 4, 5, 6, 7, 8, 9, 10, 11, 12, 13, 14, 15, 16, 17, 18, 19, 20, 21, 22, 23, 24, 25, 26, 27, 28, 29, 30, 31, 32, 33, 34, 35, 36.

Power was tested in seven study designs: unrelated individuals in case–control studies, Nuclear-family-1 and −2, Sib-pair-1 and −2, and Three-generation-1 and −2. General assumptions are a homogeneous population, 20% of causal variants, and a baseline penetrance of 0.01. Figure 2(A-D) shows the calculation of power to PCV when N = 1800 individuals.

Results from these analyses, although preliminary, confirm our hypothesis that a pedigree-based study design is more powerful than designs based on data from unrelated cases and controls, and that collapsing methods are more powerful than single-marker analysis. As expected, our results also confirm that collapsed methods without weights have weaker analytic power than either WSS or VT (although with or without weighting, differences in power are reduced with an assumed PCV as high as 20-30%), (See Figures 1, 2, 3, 4 for dominant model and Additional files 1, 2, 3, 4, 5, 6, 7, 8, 9, 10, 11, 12 for non-dominant models).

The finding that is perhaps most significant for the design of studies in future is that analytic power is directly related to both the complexity of pedigree structure and the proportion of affected individuals in the sample. We believe that the fact that more complex pedigrees contain more information on the co-inheritance of rare risk variants in association with disease status accounts for much of our proposed method’s increased power to detect rare causal variants.

This exploratory study also shows that a mixed design (Sib-pair-2, Nuclear-family-2, and Three-generation-2) is slightly less powerful than a Three-generation-2 design, and that a half-and-half mixed design (50% Sib-pair-2 and 50% Nuclear-family-2) has analytic power similar to that of the Sib-pair-2 and Nuclear-family-2 designs (See Table 3). Since mixed designs more closely approximate reality, this result increases our confidence that the proposed new method will work well with real data.

**Table 3:** Power of mixed and unmixed study designs

	**Sample Size and Power**				
Uniform Data Design					
Sib-Pair-2	900	1200	1500	1800	2100
χ^2^	0.37	0.48	0.52	0.55	0.57
Collapsing	0.51	0.58	0.62	0.66	0.69
VT	0.6	0.68	0.73	0.77	0.79
WSS	0.61	0.7	0.74	0.78	0.81
Nuclear Family 2	900	1200	1500	1800	2100
χ^2^	0.40	0.50	0.54	0.57	0.59
Collapsing	0.52	0.60	0.64	0.67	0.70
VT	0.62	0.70	0.76	0.79	0.80
WSS	0.63	0.72	0.78	0.81	0.82
Three Generation 2	900	1200	1500	1800	2100
χ^2^	0.44	0.53	0.57	0.6	0.63
Collapsing	0.54	0.62	0.67	0.7	0.73
VT	0.64	0.71	0.79	0.82	0.84
WSS	0.65	0.74	0.8	0.84	0.85
Mixed Data Designs					
Mix1 (33% Sib-Pair-2, 33% nuclear-2, and 34% Three- generation-2)	900	1200	1500	1800	2100
χ^2^	0.39	0.51	0.53	0.56	0.60
Collapsing	0.53	0.59	0.64	0.68	0.70
VT	0.62	0.68	0.73	0.77	0.82
WSS	0.62	0.69	0.75	0.81	0.84
Mix2 (50% Sib-Pair-2 and 50% Nuclear Family-2)	900	1200	1500	1800	2100
χ^2^	0.36	0.45	0.50	0.55	0.58
Collapsing	0.49	0.55	0.59	0.63	0.65
VT	0.59	0.68	0.74	0.78	0.82
WSS	0.6	0.69	0.76	0.8	0.83

According to our calculations (in which PCV varied from 10-30% and the number of sampled individuals in the pedigree varied from N = 900 to 2,100), the Three-generation-2 design consistently gives the best power, followed by Nuclear-family-2 and Sib-pair-2 designs. That is, with a power difference of approximately 4-9%, Three-generation-2 outperforms Three-generation-1; Nuclear-family-2 outperforms Nuclear-family-1; and Sib-pair-2 outperforms Three-generation-1. As expected, the case–control design gives the lowest power (See Figures 5, 6, 7, 8 and Additional files 13, 14, 15, 16, 17, 18, 19, 20, 21, 22, 23, 24).

To evaluate power where variants are associated with varying directions of association, we simulated a data set assuming that of 20% causal variants, half confer risk and half are protective. Although the presence of both risk and protective variants reduces the power to some extent, we found that the impact of opposing directions of association on power is reduced under the dominant model as the complexity of pedigree structure increases. Our method, in fact, performs best under the dominant model (see Figures 9, 10, 11, 12); has slightly reduced power under the multiplicative model, less under the additive model, and least under the recessive model (see Additional files 25, 26, 27, 28, 29, 30, 31, 32, 33, 34, 35, 36).

### Applying PB-STAR to Framingham Heart Study data set

To test our proposed study statistics on real data, we applied it to a GWAS data set from the Framingham Heart Study (FHS) [20] hosted by dbGAP. The proposed statistics were then used to test for associations of multiple variants with various cardiovascular diseases (CVD) including coronary heart disease (CHD), stroke, heart failure (HF) and atrial fibrillation (AF) (see Kannel et al. [21]).

We applied our proposed statistics to the Framingham Study data set using the Affymetrix 500 K platform, with CVD as the main phenotype. (Note that, to gain more variants with the Affymetrix 500 K platform, we changed our threshold variants from our standard 0.01 to 0.05). In this data set, a total of 1,603 individuals were genotyped, of which 267 were affected. In the end, our pedigree analysis included 462 pedigrees: 320 sib-pairs without parents, 138 pedigrees with 2 generations and 4 pedigrees with 3 generations. SNPs that failed to pass the Mendelian error check test or had allele frequencies greater than 0.05 were excluded. Our analysis included 4,376 genes with 35,507 SNPs. To obtain the estimated IBD for each pair of individuals, we randomly selected 1000 SNPs (the R-square between any pair of these SNPs was less than 0.2) spaced over the genome.

In our simulations, the WSS statistic shows consistently higher power than the other three test statistics evaluated. Using WSS with a cut-off threshold of 2 × 10^–3^, we identified 21 potentially significant genes including B4GALNT2, AKAP7, DYRK1A and FAM19A2 (See Table 4). Although the biological relationship between B4GALNT2 and human heart diseases has yet to be documented, AKAP7 [22], DYRK1A [23] and FAM19A2 [24] have all been implicated in its etiology. Taken together, these results from our analysis of FHS data support the hypothesis that the genes B4GALNT2, AKAP7 and DYRK1A may be significant for development of CVD although further molecular tests are needed to test these hypotheses although further molecular tests are warranted.

**Table 4:** P-values of four statistics for testing the association of a gene with CVD in Framingham Heart Study

**Gene**	**Number of SNPs**	**χ**^**2**^	**Collapsing**	**VT**	**WSS**
B4GALNT2	6	2.01E-03	2.10E-04	2.27E-03	6.00E-05
AKAP7	3	6.38E-02	6.61E-04	1.42E-02	1.00E-04
BOMB	5	2.48E-03	3.51E-03	8.16E-04	3.00E-04
STX11	4	1.35E-02	3.11E-03	7.78E-04	3.60E-04
PIWIL3	4	5.89E-02	8.67E-03	1.06E-02	4.50E-04
CRY1	10	5.87E-04	4.92E-01	2.84E-02	4.70E-04
PTGES3	7	3.57E-02	1.40E-02	6.42E-03	5.46E-04
HMSD	8	9.62E-03	7.65E-01	3.33E-02	8.38E-04
MNB/DYRK	9	1.02E-02	4.87E-02	3.64E-02	8.85E-04
PIK3R4	5	2.89E-03	5.51E-01	5.79E-04	1.01E-03
MAP3K5	19	7.57E-02	9.61E-02	2.36E-03	1.31E-03
ZNF823	3	2.78E-02	1.18E-03	1.58E-02	1.34E-03
CTCF	3	1.12E-01	3.83E-02	1.73E-01	1.36E-03
TRPC4	14	4.15E-02	5.99E-02	7.32E-04	1.50E-03
OSBPL9	12	9.09E-03	1.45E-04	1.83E-02	1.53E-03
DYRK1A	12	1.47E-02	7.78E-02	3.47E-02	1.58E-03
FAM19A2	13	2.65E-01	2.28E-03	9.43E-03	1.60E-03
MRPS18C	12	2.19E-03	5.37E-03	2.51E-03	1.63E-03
FAM175A	9	2.43E-03	3.51E-03	2.11E-03	1.67E-03
ZNF714	6	3.40E-03	1.16E-02	2.39E-03	1.85E-03
AGPAT5	9	1.96E-02	1.68E-01	6.85E-03	1.94E-03

## Discussion

While a number of methods currently exist for collapsing rare variants into a single group to test for differences in their collective frequency in cases and controls, methods using family-based statistics to test for rare variants associations in multi-generational families have rarely been discussed. Since we expect causal rare variants to be more enriched in extended pedigrees than in the general population and also in nuclear families, complex pedigrees should be the ideal source of information on rare variants’ contribution to human disorders. Results from our preliminary simulations appear to support the added value of looking for rare causal genetic variants in large and complex pedigrees.

As described in the Methods and Results sections above, we devised simulations to test the power of our new statistics and their type I error rates. Results from tests using seven different study designs and dominant, additive, recessive, and multiplicative models of disease indicate that our statistic performs best with the dominant disease model and, as expected, a study population made up of three-generation families with an affected/ unaffected ratio of 2 to 1.

These results suggest that our proposed statistics can substantially benefit researchers seeking to sequence exomes or whole genomes with a pedigree-based approach. Since computations based on family data association tests are almost as efficient as those based on population data, moreover, it should be possible to combine results from both. (See, for instance, Table 3, which contains results from pedigree-based association tests to detect rare variants in mixed-pedigree populations.)

Additionally, while earlier family-based linkage approaches rely on chromosomal segments shared by related individuals within pedigrees, our method reveals nucleotide-site similarities in segments shared across pedigrees.

As indicated in our introduction, this work was inspired by Thornton and McPeek [25] who offer two ways to analyze genetic associations: 1) using the standard χ^2^ statistic with a correction factor that takes pedigree information into account; and 2) using a factor that corrects for the conditional probability of IBD sharing. In a later publication [16], the same authors proposed the “Quasi-likelihood Score” (W_QLS_), another useful statistic that, according to their simulations, outperforms earlier methods. The new method introduced here uses a correction method (detailed in the Method section above) similar to that of Thornton and McPeek. While earlier pedigree-based methods are limited to the analysis of single markers, ours analyzes associations among multiple markers. Our results confirm the superior power of family-based analysis. They also confirm the need to correct for relatedness in order to reach appropriate rates of type I error.

Before drawing conclusions from this study, we would like to point out its limitation. As a ‘proof of concept’ analysis for a new statistic for the analysis of pedigree data, this study is of necessity schematic and introductory. In our simulations, for instance, both disease models and population structures were purposefully kept simple enough for us to monitor statistical behavior. Although our results are preliminary, they appear to confirm the new test statistic’s potential usefulness for the analysis of pedigree-based NGS data.

## Conclusions

This study introduces a new, family-based statistic to analyze for rare variants segregated in pedigrees. This new statistic is based on three principles: 1) It collapses data to deal with the problem of identifying rare variants in a gene or a genomic region. 2) It uses IBD coefficients to correct for relatedness and assure validity and power. 3) It applies two weights, WSS and VT, to increase the statistic’s power to detect rare variants.

Using computer simulations, we showed that 1) our pedigree-based design is more powerful than population based case–control designs; 2) the higher the number of affected individuals in a pedigree, the higher the complement of rare variants 3) WSS performs slightly better than VT; and 4) as the proportion of causal variants increases, so does the power gain of WSS or VT over an un-weighted collapsing method. The power gain using WSS and VT versus the collapsing method without weights increases with the increase in proportion of causal variants. Finally, we confirmed the usefulness of our new statistic in real data, a GWAS data set from the FHS. Since NGS data from the same cohort are expected to be available soon on the genes containing rare variants associated with heart disease identified by our analysis, we look forward to being able to use these data to validate our current findings, and to discover new signals, in the near future. Our “PB-STAR” software is now freely available at: https://sph.uth.edu/hgc/faculty/xiong/software-E.html.

## Competing interests

The authors declare that they have no competing interests.

## Authors’ contributions

YYS, MX, YZ and WG all contributed to the study design, analytical preparation, and simulation modeling. MX contributed to the derivations, YZ conducted all calculations of type I error rates and power. All four authors participated in strategic planning, concept development, revisions, and manuscript preparation. All authors read and approved the final manuscript.

## Supplementary Material

Additional file 1: Figure S1A The power curves of the family-based corrected single marker χ^2^ test statistic as a function of the total number of individuals at the significance level α = 0.05 in the test under seven settings: unrelated individuals in cases-controls study, nuclear family groups 1 and 2, sib-pair groups 1 and 2 and three generation family groups 1 and 2, assuming an additive model, 20% of the risk variants and a baseline penetrance of 0.01. Click here for file

Additional file 2: Figure S1B The power curves of the family-based collapsing test (variants with frequencies ≤0.005 were collapsed) statistic as a function of the total number of individuals at the significance level α = 0.05 in the test under seven settings: unrelated individuals in cases-controls study, nuclear family groups 1 and 2, sib-pair groups 1 and 2 and three generation family groups 1 and 2, assuming an additive model, 20% of the risk variants and a baseline penetrance of 0.01. Click here for file

Additional file 3: Figure S1C The power curves of the family-based VT test statistic as a function of the total number of individuals at the significance level α = 0.05 in the test under seven settings: unrelated individuals in cases-controls study, nuclear family groups 1 and 2, sib-pair groups 1 and 2 and three generation family groups 1 and 2, assuming a dominant model, 20% of the risk variants and a baseline penetrance of 0.01. Click here for file

Additional file 4: Figure S1D The power curves of the family-based WSS test statistic as a function of the total number of individuals at the significance level α = 0.05 in the test under seven settings: unrelated individuals in cases-controls study, nuclear family groups 1 and 2, sib-pair groups 1 and 2 and three generation family groups 1 and 2, assuming an additive model, 20% of the risk variants and a baseline penetrance of 0.01. Click here for file

Additional file 5: Figure S2A The power curves of the family-based corrected single marker χ^2^ test statistic as a function of the total number of individuals at the significance level α = 0.05 in the test under seven settings: unrelated individuals in cases-controls study, nuclear family groups 1 and 2, sib-pair groups 1 and 2 and three generation family groups 1 and 2, assuming a multiplicative model, 20% of the risk variants and a baseline penetrance of 0.01. Click here for file

Additional file 6: Figure S2B The power curves of the family-based collapsing test (variants with frequencies ≤0.005 were collapsed) statistic as a function of the total number of individuals at the significance level α = 0.05 in the test under seven settings: unrelated individuals in cases-controls study, nuclear family groups 1 and 2, sib-pair groups 1 and 2 and three generation family groups 1 and 2, assuming a multiplicative model, 20% of the risk variants and a baseline penetrance of 0.01. Click here for file

Additional file 7: Figure S2C The power curves of the family-based VT test statistic as a function of the total number of individuals at the significance level α = 0.05 in the test under seven settings: unrelated individuals in cases-controls study, nuclear family groups 1 and 2, sib-pair groups 1 and 2 and three generation family groups 1 and 2, assuming a multiplicative model, 20% of the risk variants and a baseline penetrance of 0.01. Click here for file

Additional file 8: Figure S2D The power curves of the family-based WSS test statistic as a function of the total number of individuals at the significance level α = 0.05 in the test under seven settings: unrelated individuals in cases-controls study, nuclear family groups 1 and 2, sib-pair groups 1 and 2 and three generation family groups 1 and 2, assuming a multiplicative model, 20% of the risk variants and a baseline penetrance of 0.01. Click here for file

Additional file 9: Figure S3A The power curves of the family-based corrected single marker χ^2^ test statistic as a function of the total number of individuals at the significance level α = 0.05 in the test under seven settings: unrelated individuals in cases-controls study, nuclear family groups 1 and 2, sib-pair groups 1 and 2 and three generation family groups 1 and 2, assuming a recessive model, 20% of the risk variants and a baseline penetrance of 0.01. Click here for file

Additional file 10: Figure S3B The power curves of the family-based collapsing test (variants with frequencies ≤0.005 were collapsed) statistic as a function of the total number of individuals at the significance level α = 0.05 in the test under seven settings: unrelated individuals in cases-controls study, nuclear family groups 1 and 2, sib-pair groups 1 and 2 and three generation family groups 1 and 2, assuming a recessive model, 20% of the risk variants and a baseline penetrance of 0.01. Click here for file

Additional file 11: Figure S3C The power curves of the family-based VT test statistic as a function of the total number of individuals at the significance level α = 0.05 in the test under seven settings: unrelated individuals in cases-controls study, nuclear family groups 1 and 2, sib-pair groups 1 and 2 and three generation family groups 1 and 2, assuming a recessive model, 20% of the risk variants and a baseline penetrance of 0.01. Click here for file

Additional file 12: Figure S3D The power curves of the family-based WSS test statistic as a function of the total number of individuals at the significance level α = 0.05 in the test under seven settings: unrelated individuals in cases-controls study, nuclear family groups 1 and 2, sib-pair groups 1 and 2 and three generation family groups 1 and 2, assuming a recessive model, 20% of the risk variants and a baseline penetrance of 0.01. Click here for file

Additional file 13: Figure 4A The power curves of the family-based corrected single marker χ^2^ test statistic as a function of the proportion of risk variants at the significance level α = 0.05 in the test under seven settings: unrelated individuals in cases-controls study, nuclear family groups 1 and 2, sib-pair groups 1 and 2 and three generation family groups 1 and 2, assuming an additive model, a total of 1,800 sampled individuals and a baseline penetrance of 0.01. Click here for file

Additional file 14: Figure 4B The power curves of the family-based collapsing test (variants with frequencies _≤_0.005 were collapsed) statistic as a function of the proportion of risk variants at the significance level α = 0.05 in the test under seven settings: unrelated individuals in cases-controls study, nuclear family groups 1 and 2, sib-pair groups 1 and 2 and three generation family groups 1 and 2, assuming an additive model, a total of 1,800 sampled individuals and a baseline penetrance of 0.01. Click here for file

Additional file 15: Figure 4C The power curves of the family-based VT test statistic as a function of the proportion of risk variants at the significance level α = 0.05 in the test under seven settings: unrelated individuals in cases-controls study, nuclear family groups 1 and 2, sib-pair groups 1 and 2 and three generation family groups 1 and 2, assuming an additive model, a total of 1,800 sampled individuals and a baseline penetrance of 0.01. Click here for file

Additional file 16: Figure 4D The power curves of the family-based WSS test statistic as a function of the proportion of risk variants at the significance level α = 0.05 in the test under seven settings: unrelated individuals in cases-controls study, nuclear family groups 1 and 2, sib-pair groups 1 and 2 and three generation family groups 1 and 2, assuming an additive model, a total of 1,800 sampled individuals and a baseline penetrance of 0.01. Click here for file

Additional file 17: Figure S5A The power curves of the family-based corrected single marker χ^2^ test statistic as a function of the proportion of risk variants at the significance level α = 0.05 in the test under seven settings: unrelated individuals in cases-controls study, nuclear family groups 1 and 2, sib-pair groups 1 and 2 and three generation family groups 1 and 2, assuming a multiplicative model, a total of 1,800 sampled individuals and a baseline penetrance of 0.01. Click here for file

Additional file 18: Figure S5B The power curves of the family-based collapsing test (variants with frequencies ≤0.005 were collapsed) statistic as a function of the proportion of risk variants at the significance level α = 0.05 in the test under seven settings: unrelated individuals in cases-controls study, nuclear family groups 1 and 2, sib-pair groups 1 and 2 and three generation family groups 1 and 2, assuming a multiplicative model, a total of 1,800 sampled individuals and a baseline penetrance of 0.01. Click here for file

Additional file 19: Figure S5C The power curves of the family-based VT test statistic as a function of the proportion of risk variants at the significance level α = 0.05 in the test under seven settings: unrelated individuals in cases-controls study, nuclear family groups 1 and 2, sib-pair groups 1 and 2 and three generation family groups 1 and 2, assuming the multiplicative model, a total of 1,800 sampled individuals and a baseline penetrance of 0.01. Click here for file

Additional file 20: Figure S5D The power curves of the family-based WSS test statistic as a function of the proportion of risk variants at the significance level α = 0.05 in the test under seven settings: unrelated individuals in cases-controls study, nuclear family groups 1 and 2, sib-pair groups 1 and 2 and three generation family groups 1 and 2, assuming the multiplicative model, a total of 1,800 sampled individuals and a baseline penetrance of 0.01. Click here for file

Additional file 21: Figure S6A The power curves of the family-based corrected single marker χ^2^ test statistic as a function of the proportion of risk variants at the significance level α = 0.05 in the test under seven settings: unrelated individuals in cases-controls study, nuclear family groups 1 and 2, sib-pair groups 1 and 2 and three generation family groups 1 and 2, assuming a recessive model, a total of 1,800 sampled individuals and a baseline penetrance of 0.01. Click here for file

Additional file 22: Figure S6B The power curves of the family-based collapsing test (variants with frequencies ≤0.005 were collapsed) statistic as a function of the proportion of risk variants at the significance level α = 0.05 in the test under seven settings: unrelated individuals in cases-controls study, nuclear family groups 1 and 2, sib-pair groups 1 and 2 and three generation family groups 1 and 2, assuming a recessive model, a total of 1,800 sampled individuals and a baseline penetrance of 0.01. Click here for file

Additional file 23: Figure S6C The power curves of the family-based VT test statistic as a function of the proportion of risk variants at the significance level α = 0.05 in the test under seven settings: unrelated individuals in cases-controls study, nuclear family groups 1 and 2, sib-pair groups 1 and 2 and three generation family groups 1 and 2, assuming the recessive model, a total of 1,800 sampled individuals and a baseline penetrance of 0.01. Click here for file

Additional file 24: Figure S6D The power curves of the family-based WSS test statistic as a function of the proportion of risk variants at the significance level α = 0.05 in the test under seven settings: unrelated individuals in cases-controls study, nuclear family groups 1 and 2, sib-pair groups 1 and 2 and three generation family groups 1 and 2, assuming the recessive model, a total of 1,800 sampled individuals and a baseline penetrance of 0.01. Click here for file

Additional file 25: Figure S7A The power curves of the family-based corrected single marker χ^2^ statistic under opposite directions of association as a function of the total number of individuals at the significance level α = 0.05 in the test under seven settings: unrelated individuals in cases-controls study, nuclear family groups 1 and 2, sib-pair groups 1 and 2 and three generation family groups 1 and 2, assuming an additive model, 20% of the risk variants and a baseline penetrance of 0.01. Click here for file

Additional file 26: Figure S7B The power curves of the family-based collapsing test (variants with frequencies ≤0.005 were collapsed) statistic under opposite directions of association as a function of the total number of individuals at the significance level α = 0.05 in the test under seven settings: unrelated individuals in cases-controls study, nuclear family groups 1 and 2, sib-pair groups 1 and 2 and three generation family groups 1 and 2, assuming an additive model, 20% of the risk variants and a baseline penetrance of 0.01. Click here for file

Additional file 27: Figure S7C The power curves of the family-based VT statistic under opposite directions of association as a function of the total number of individuals at the significance level α = 0.05 in the test under seven settings: unrelated individuals in cases-controls study, nuclear family groups 1 and 2, sib-pair groups 1 and 2 and three generation family groups 1 and 2, assuming an additive model, 20% of the risk variants and a baseline penetrance of 0.01. Click here for file

Additional file 28: Figure S7D The power curves of the family-based WSS test statistic under opposite directions of association as a function of the total number of individuals at the significance level α = 0.05 in the test under seven settings: unrelated individuals in cases-controls study, nuclear family groups 1 and 2, sib-pair groups 1 and 2 and three generation family groups 1 and 2, assuming an additive model, 20% of the risk variants and a baseline penetrance of 0.01. Click here for file

Additional file 29: Figure S8A The power curves of the family-based corrected single marker χ^2^ statistic under opposite directions of association as a function of the total number of individuals at the significance level α = 0.05 in the test under seven settings: unrelated individuals in cases-controls study, nuclear family groups 1 and 2, sib-pair groups 1 and 2 and three generation family groups 1 and 2, assuming a multiplicative model, 20% of the risk variants and a baseline penetrance of 0.01. Click here for file

Additional file 30: Figure S8B The power curves of the family-based collapsing test (variants with frequencies ≤0.005 were collapsed) statistic under opposite directions of association as a function of the total number of individuals at the significance level α = 0.05 in the test under seven settings: unrelated individuals in cases-controls study, nuclear family groups 1 and 2, sib-pair groups 1 and 2 and three generation family groups 1 and 2, assuming a multiplicative model, 20% of the risk variants and a baseline penetrance of 0.01. Click here for file

Additional file 31: Figure S8C The power curves of the family-based VT statistic under opposite directions of association as a function of the total number of individuals at the significance level α = 0.05 in the test under seven settings: unrelated individuals in cases-controls study, nuclear family groups 1 and 2, sib-pair groups 1 and 2 and three generation family groups 1 and 2, assuming a multiplicative model, 20% of the risk variants and a baseline penetrance of 0.01. Click here for file

Additional file 32: Figure S8D The power curves of the family-based WSS test statistic under opposite directions of association as a function of the total number of individuals at the significance level α = 0.05 in the test under seven settings: unrelated individuals in cases-controls study, nuclear family groups 1 and 2, sib-pair groups 1 and 2 and three generation family groups 1 and 2, assuming a multiplicative model, 20% of the risk variants and a baseline penetrance of 0.01. (PDF 4 kb) (PDF 4 kb) Click here for file

Additional file 33: Figure S9A The power curves of the family-based corrected single marker χ^2^ statistic under opposite directions of association as a function of the total number of individuals at the significance level α = 0.05 in the test under seven settings: unrelated individuals in cases-controls study, nuclear family groups 1 and 2, sib-pair groups 1 and 2 and three generation family groups 1 and 2, assuming a recessive model, 20% of the risk variants and a baseline penetrance of 0.01. Click here for file

Additional file 34: Figure S9B The power curves of the family-based collapsing test (variants with frequencies ≤0.005 were collapsed) statistic under opposite directions of association as a function of the total number of individuals at the significance level α = 0.05 in the test under seven settings: unrelated individuals in cases-controls study, nuclear family groups 1 and 2, sib-pair groups 1 and 2 and three generation family groups 1 and 2, assuming a recessive model, 20% of the risk variants and a baseline penetrance of 0.01. Click here for file

Additional file 35: Figure S9C The power curves of the family-based VT statistic under opposite directions of association as a function of the total number of individuals at the significance level α = 0.05 in the test under seven settings: unrelated individuals in cases-controls study, nuclear family groups 1 and 2, sib-pair groups 1 and 2 and three generation family groups 1 and 2, assuming a recessive model, 20% of the risk variants and a baseline penetrance of 0.01. Click here for file

Additional file 36: Figure S9D The power curves of the family-based WSS test statistic under opposite directions of association as a function of the total number of individuals at the significance level α = 0.05 in the test under seven settings: unrelated individuals in cases-controls study, nuclear family groups 1 and 2, sib-pair groups 1 and 2 and three generation family groups 1 and 2, assuming a recessive model, 20% of the risk variants and a baseline penetrance of 0.01. Click here for file
