# Supplementary figures and images for: Weighted pedigree-based statistics for testing the association of rare variants
Source: BMC Genomics. 2012 Nov 24;13:667. doi: 10.1186/1471-2164-13-667 (PMC3827928; doi:10.1186/1471-2164-13-667)

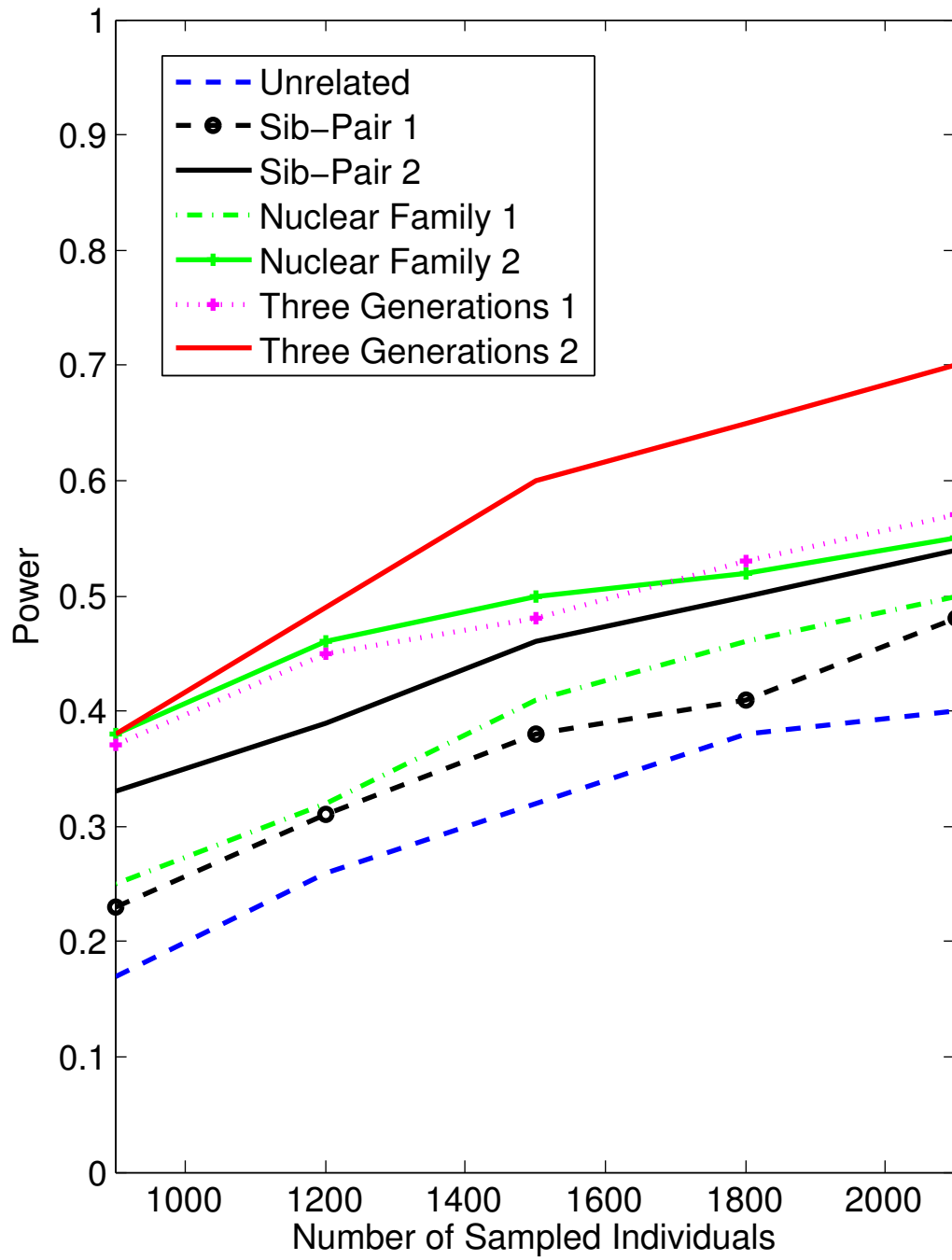

Figure 5

Supplement: Additional file 1: Figure S1A — The power curves of the family-based corrected single marker χ2 test statistic as a function of the total number of individuals at the significance level α = 0.05 in the test under seven settings: unrelated individuals in cases-controls study, nuclear family groups 1 and 2, sib-pair groups 1 and 2 and three generation family groups 1 and 2, assuming an additive model, 20% of the risk variants and a baseline penetrance of 0.01. [file 1471-2164-13-667-S1.pdf]

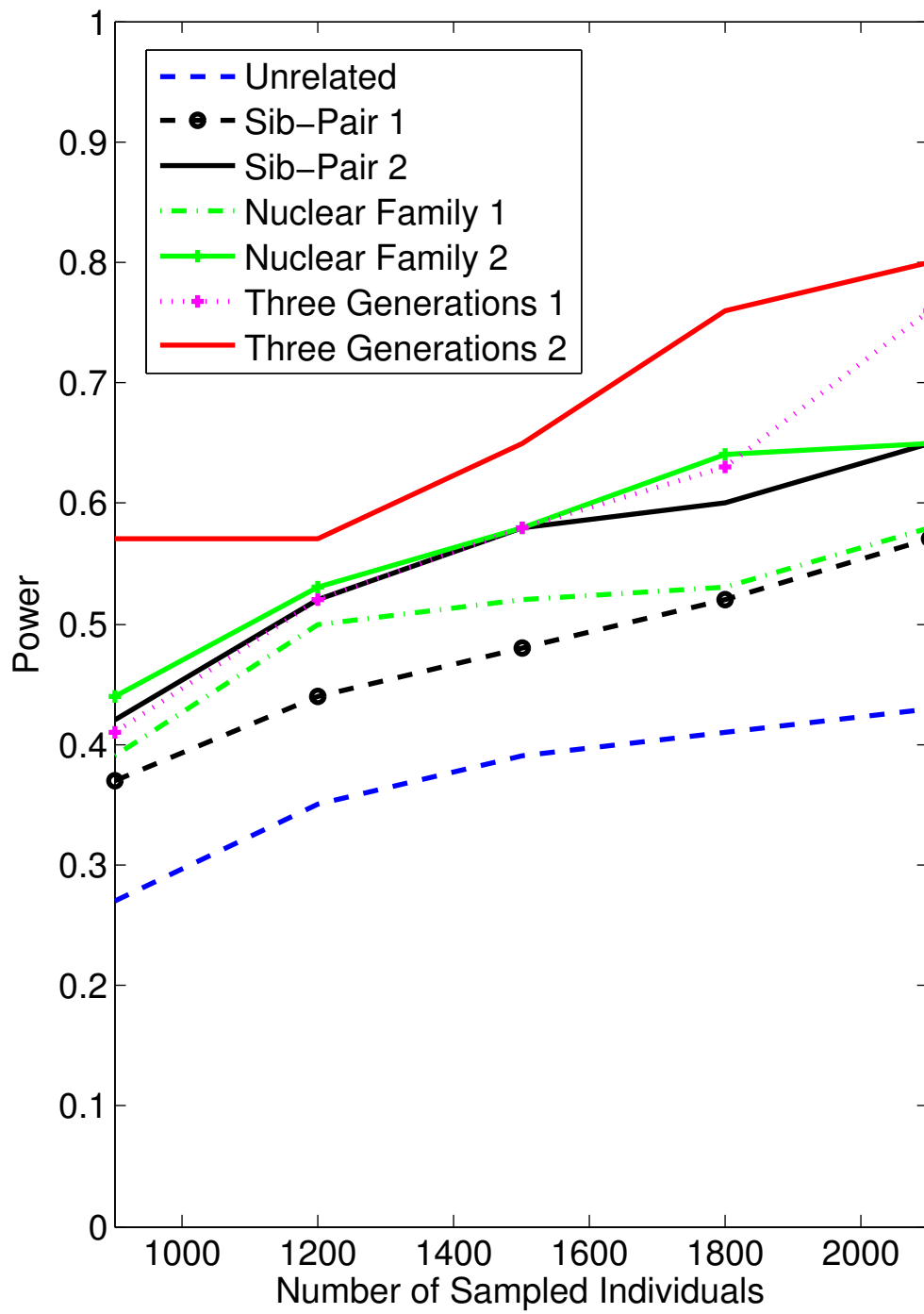

Figure 6

Supplement: Additional file 2: Figure S1B — The power curves of the family-based collapsing test (variants with frequencies ≤0.005 were collapsed) statistic as a function of the total number of individuals at the significance level α = 0.05 in the test under seven settings: unrelated individuals in cases-controls study, nuclear family groups 1 and 2, sib-pair groups 1 and 2 and three generation family groups 1 and 2, assuming an additive model, 20% of the risk variants and a baseline penetrance of 0.01. [file 1471-2164-13-667-S2.pdf]

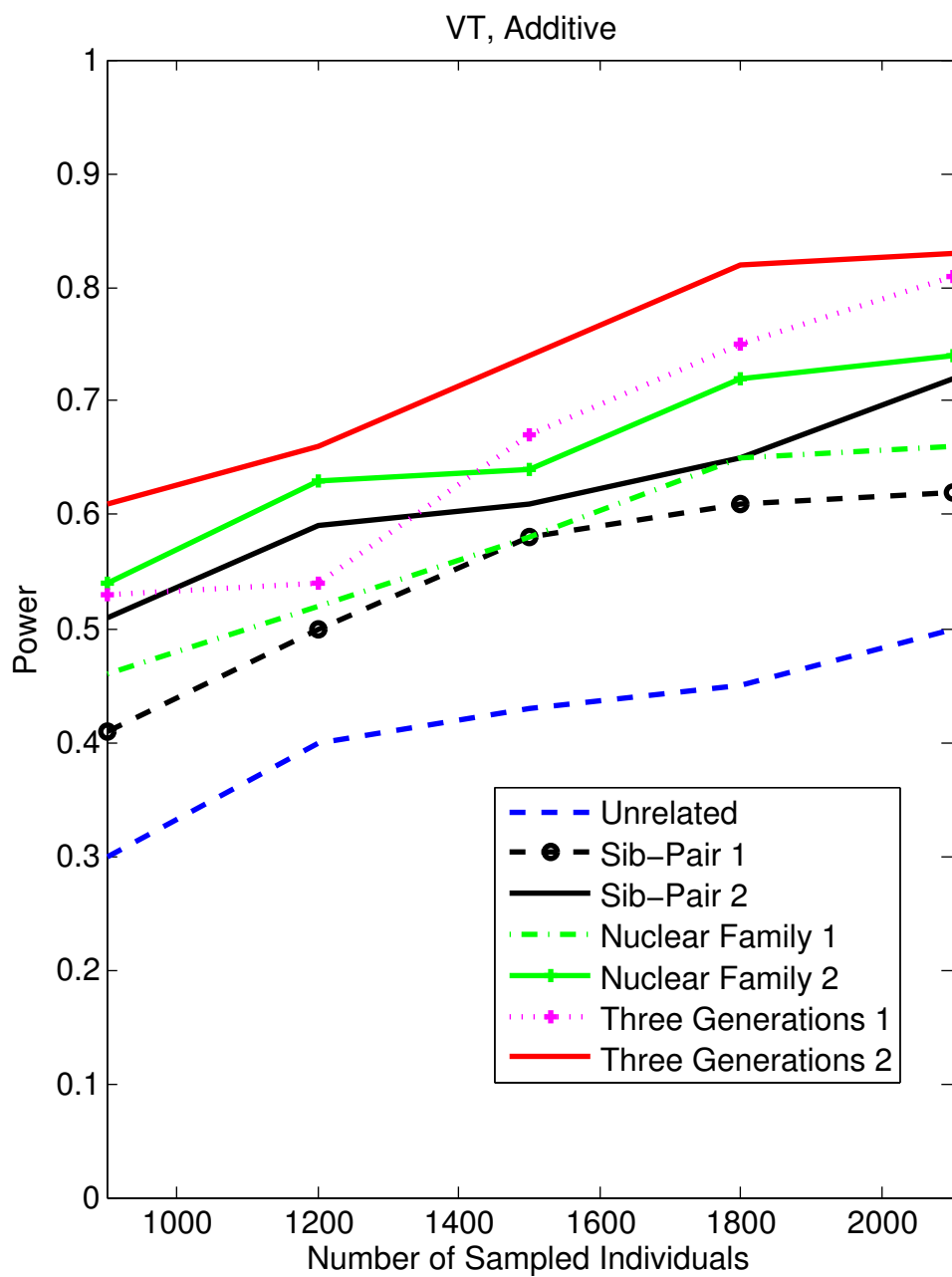

Figure 7

Supplement: Additional file 3: Figure S1C — The power curves of the family-based VT test statistic as a function of the total number of individuals at the significance level α = 0.05 in the test under seven settings: unrelated individuals in cases-controls study, nuclear family groups 1 and 2, sib-pair groups 1 and 2 and three generation family groups 1 and 2, assuming a dominant model, 20% of the risk variants and a baseline penetrance of 0.01. [file 1471-2164-13-667-S3.pdf]

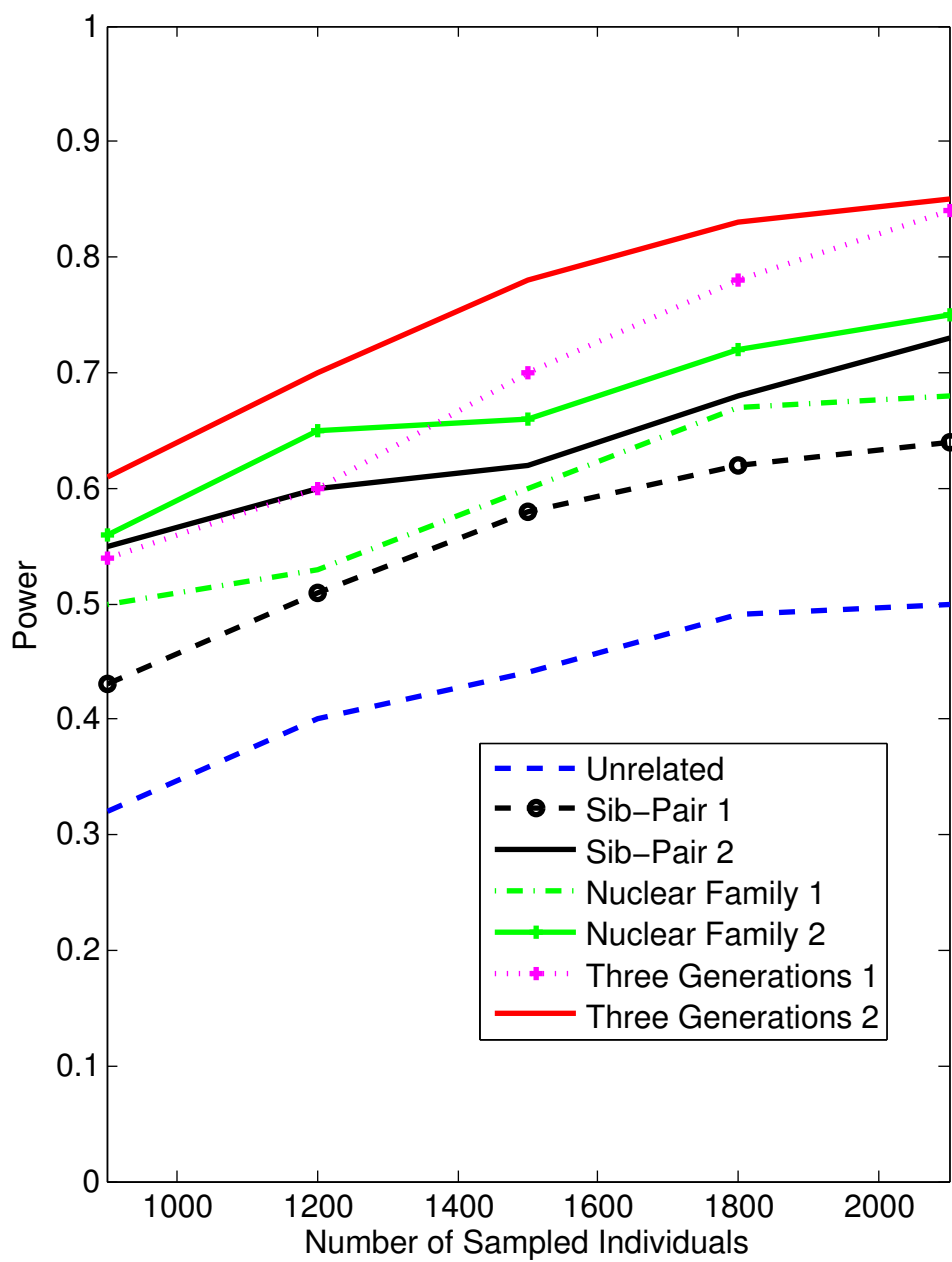

Figure 8

Supplement: Additional file 4: Figure S1D — The power curves of the family-based WSS test statistic as a function of the total number of individuals at the significance level α = 0.05 in the test under seven settings: unrelated individuals in cases-controls study, nuclear family groups 1 and 2, sib-pair groups 1 and 2 and three generation family groups 1 and 2, assuming an additive model, 20% of the risk variants and a baseline penetrance of 0.01. [file 1471-2164-13-667-S4.pdf]

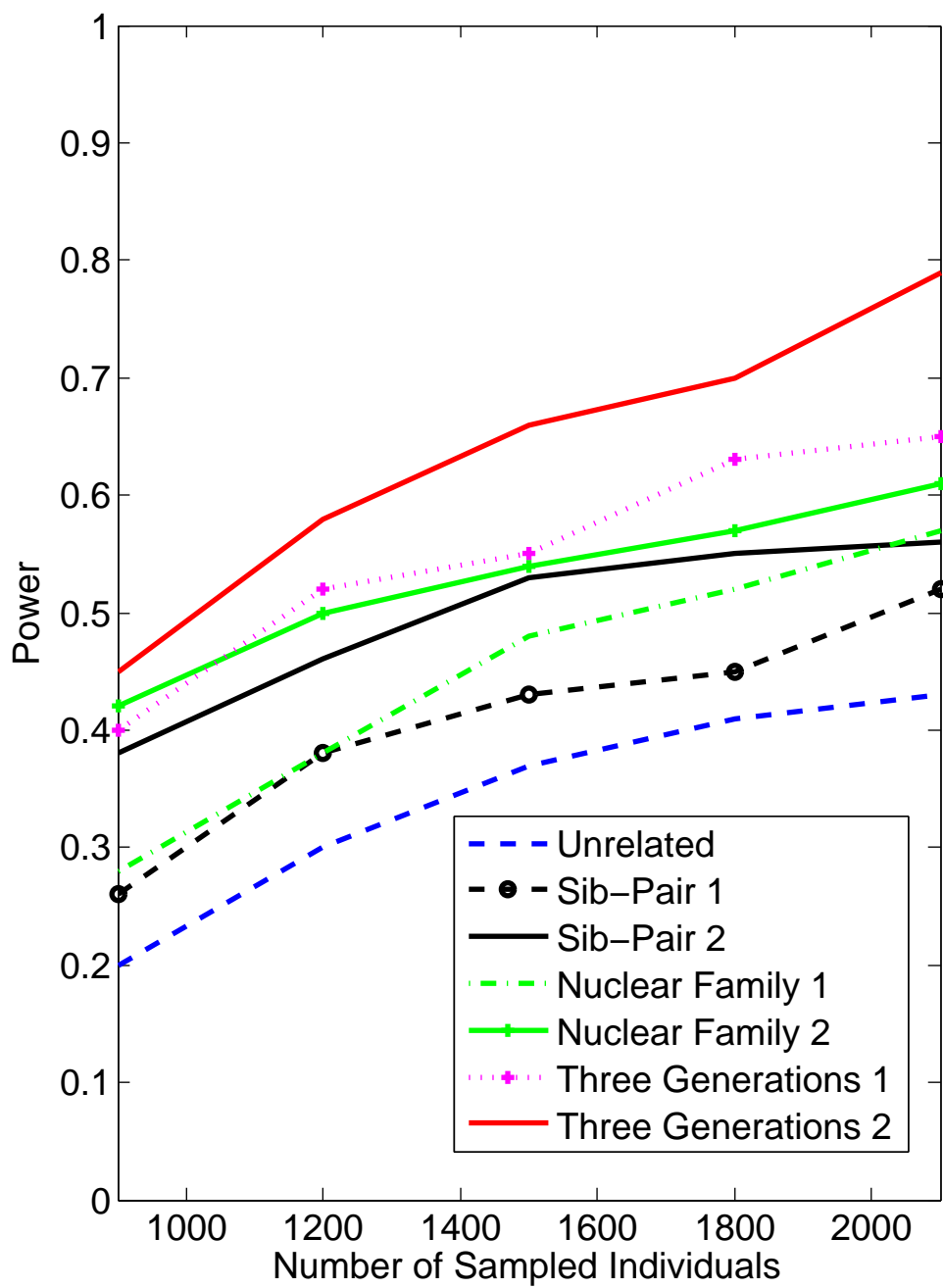

Supplement: Additional file 5: Figure S2A — The power curves of the family-based corrected single marker χ2 test statistic as a function of the total number of individuals at the significance level α = 0.05 in the test under seven settings: unrelated individuals in cases-controls study, nuclear family groups 1 and 2, sib-pair groups 1 and 2 and three generation family groups 1 and 2, assuming a multiplicative model, 20% of the risk variants and a baseline penetrance of 0.01. [file 1471-2164-13-667-S5.pdf]

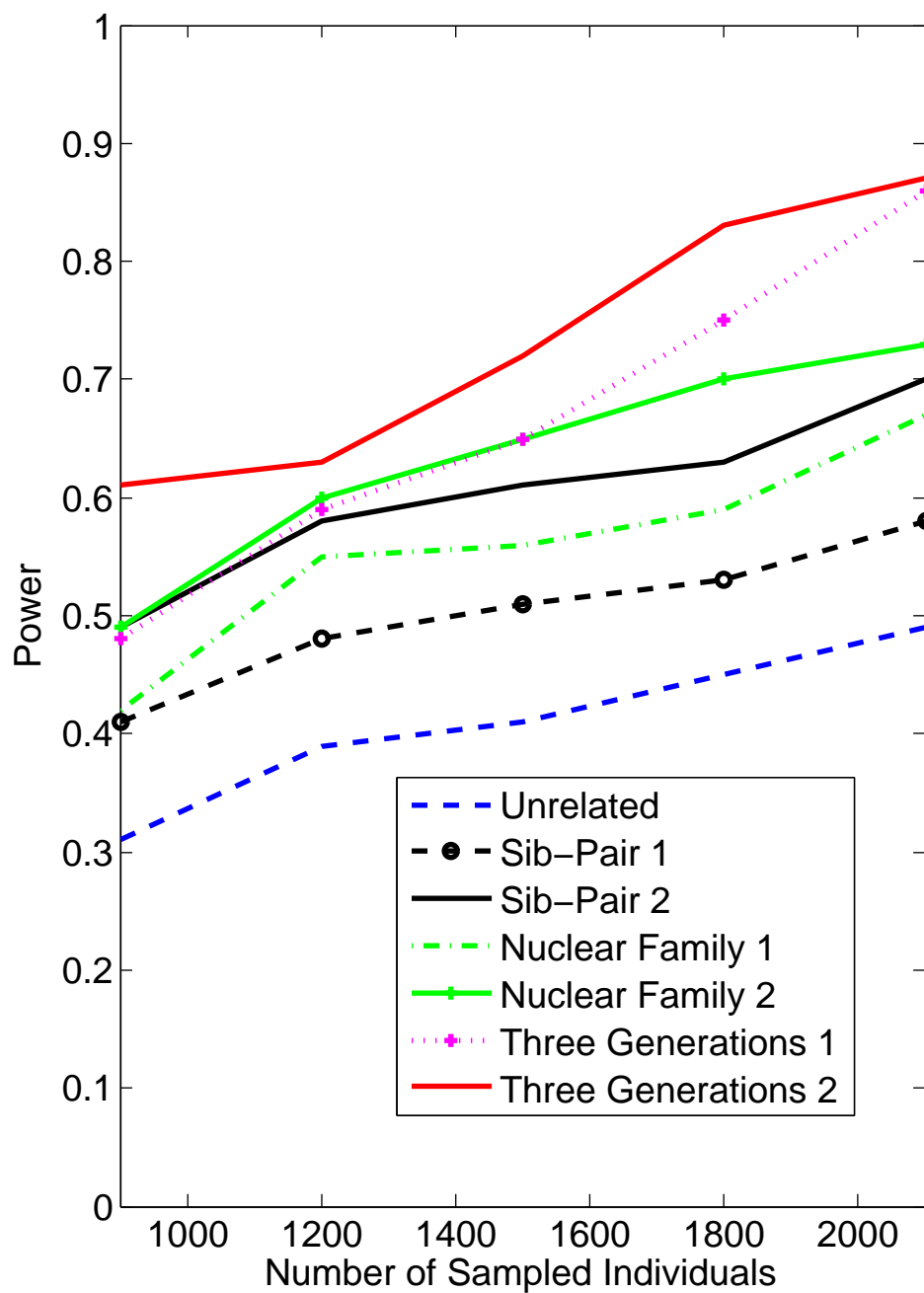

Supplement: Additional file 6: Figure S2B — The power curves of the family-based collapsing test (variants with frequencies ≤0.005 were collapsed) statistic as a function of the total number of individuals at the significance level α = 0.05 in the test under seven settings: unrelated individuals in cases-controls study, nuclear family groups 1 and 2, sib-pair groups 1 and 2 and three generation family groups 1 and 2, assuming a multiplicative model, 20% of the risk variants and a baseline penetrance of 0.01. [file 1471-2164-13-667-S6.pdf]

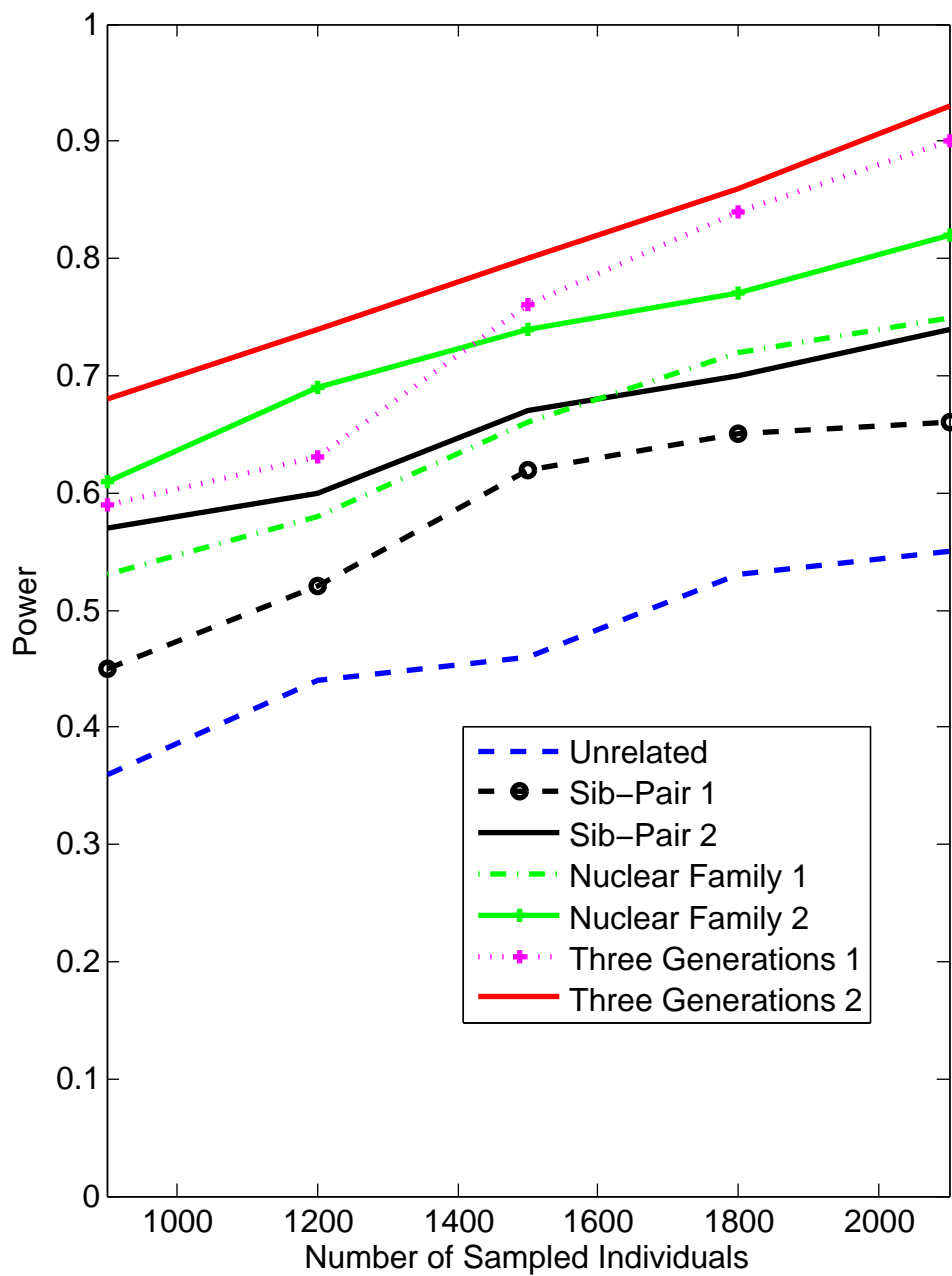

Supplement: Additional file 7: Figure S2C — The power curves of the family-based VT test statistic as a function of the total number of individuals at the significance level α = 0.05 in the test under seven settings: unrelated individuals in cases-controls study, nuclear family groups 1 and 2, sib-pair groups 1 and 2 and three generation family groups 1 and 2, assuming a multiplicative model, 20% of the risk variants and a baseline penetrance of 0.01. [file 1471-2164-13-667-S7.pdf]

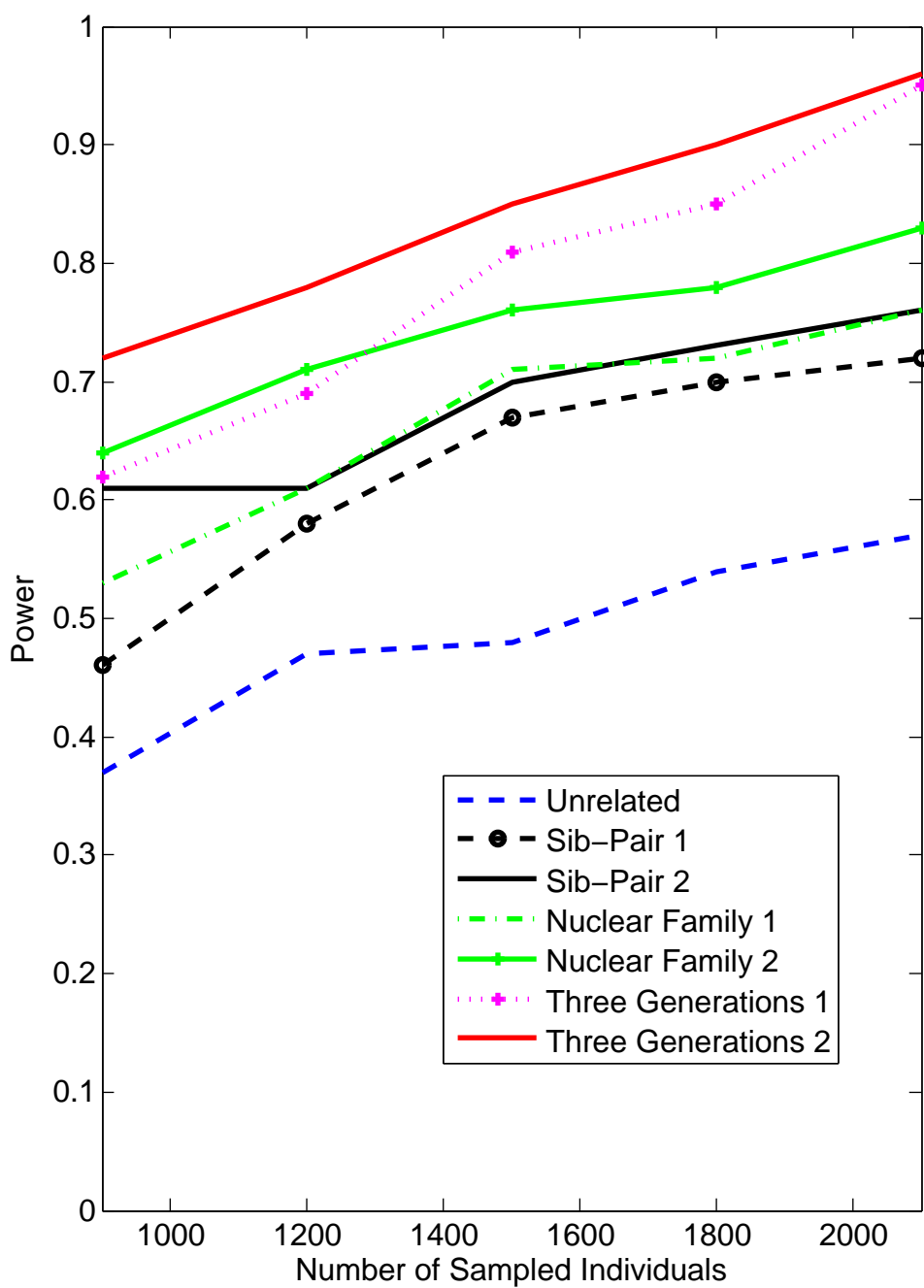

Supplement: Additional file 8: Figure S2D — The power curves of the family-based WSS test statistic as a function of the total number of individuals at the significance level α = 0.05 in the test under seven settings: unrelated individuals in cases-controls study, nuclear family groups 1 and 2, sib-pair groups 1 and 2 and three generation family groups 1 and 2, assuming a multiplicative model, 20% of the risk variants and a baseline penetrance of 0.01. [file 1471-2164-13-667-S8.pdf]

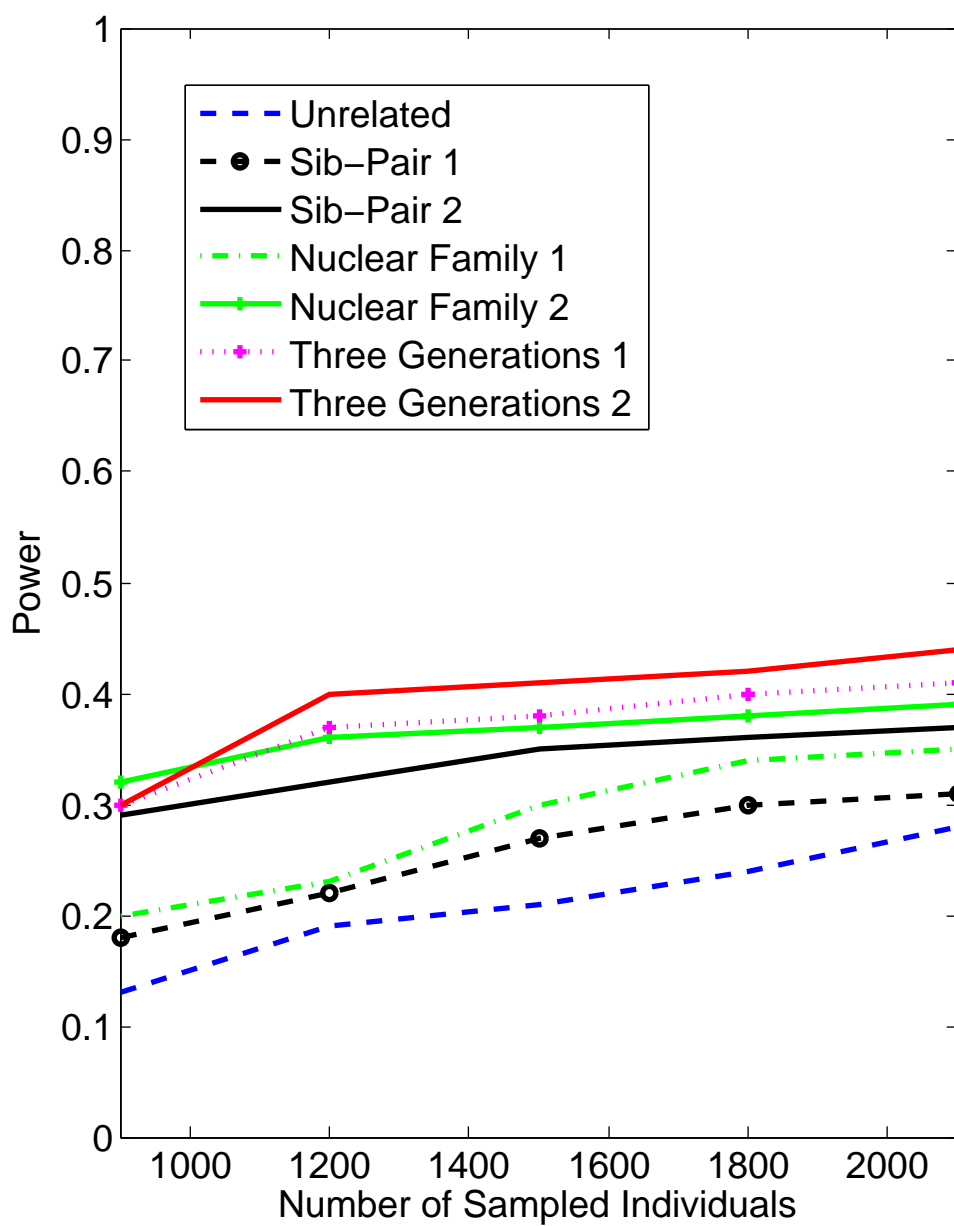

Supplement: Additional file 9: Figure S3A — The power curves of the family-based corrected single marker χ2 test statistic as a function of the total number of individuals at the significance level α = 0.05 in the test under seven settings: unrelated individuals in cases-controls study, nuclear family groups 1 and 2, sib-pair groups 1 and 2 and three generation family groups 1 and 2, assuming a recessive model, 20% of the risk variants and a baseline penetrance of 0.01. [file 1471-2164-13-667-S9.pdf]

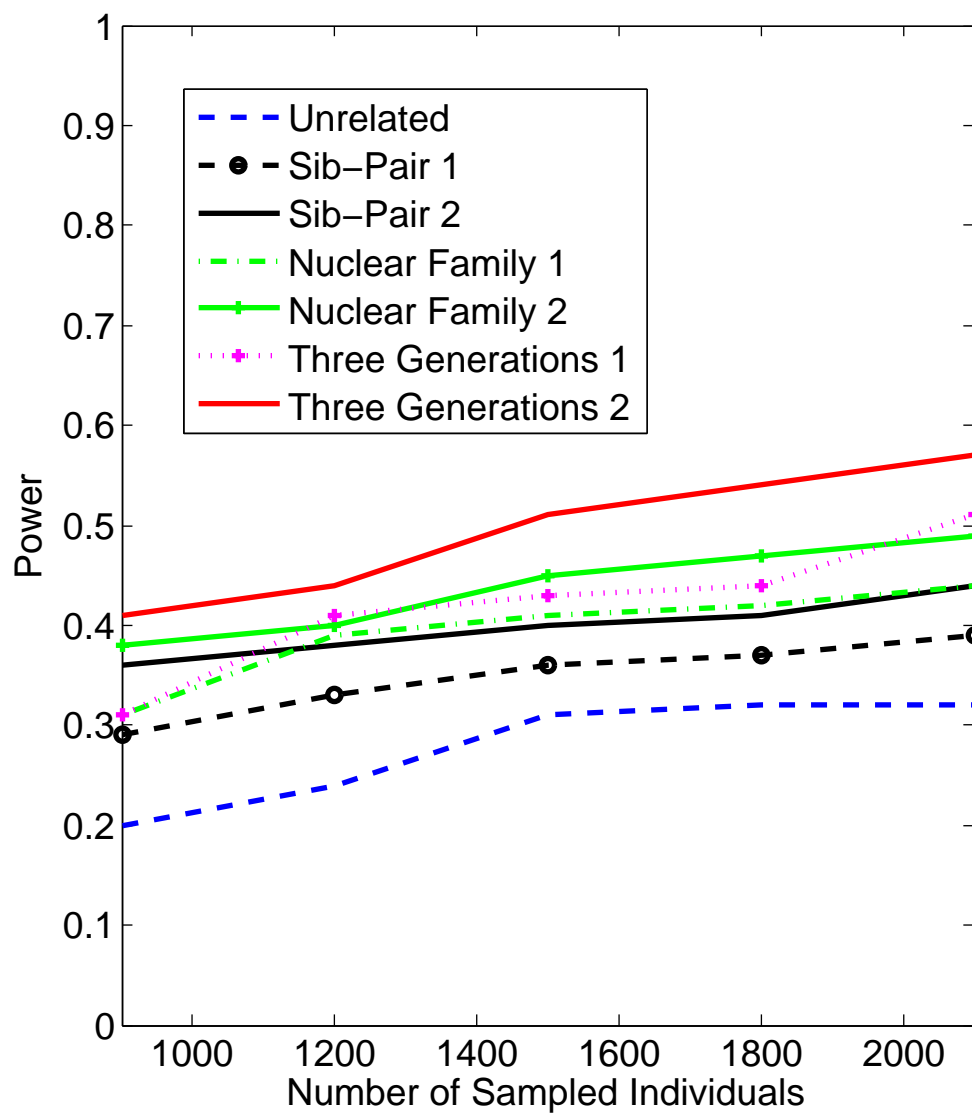

Supplement: Additional file 10: Figure S3B — The power curves of the family-based collapsing test (variants with frequencies ≤0.005 were collapsed) statistic as a function of the total number of individuals at the significance level α = 0.05 in the test under seven settings: unrelated individuals in cases-controls study, nuclear family groups 1 and 2, sib-pair groups 1 and 2 and three generation family groups 1 and 2, assuming a recessive model, 20% of the risk variants and a baseline penetrance of 0.01. [file 1471-2164-13-667-S10.pdf]

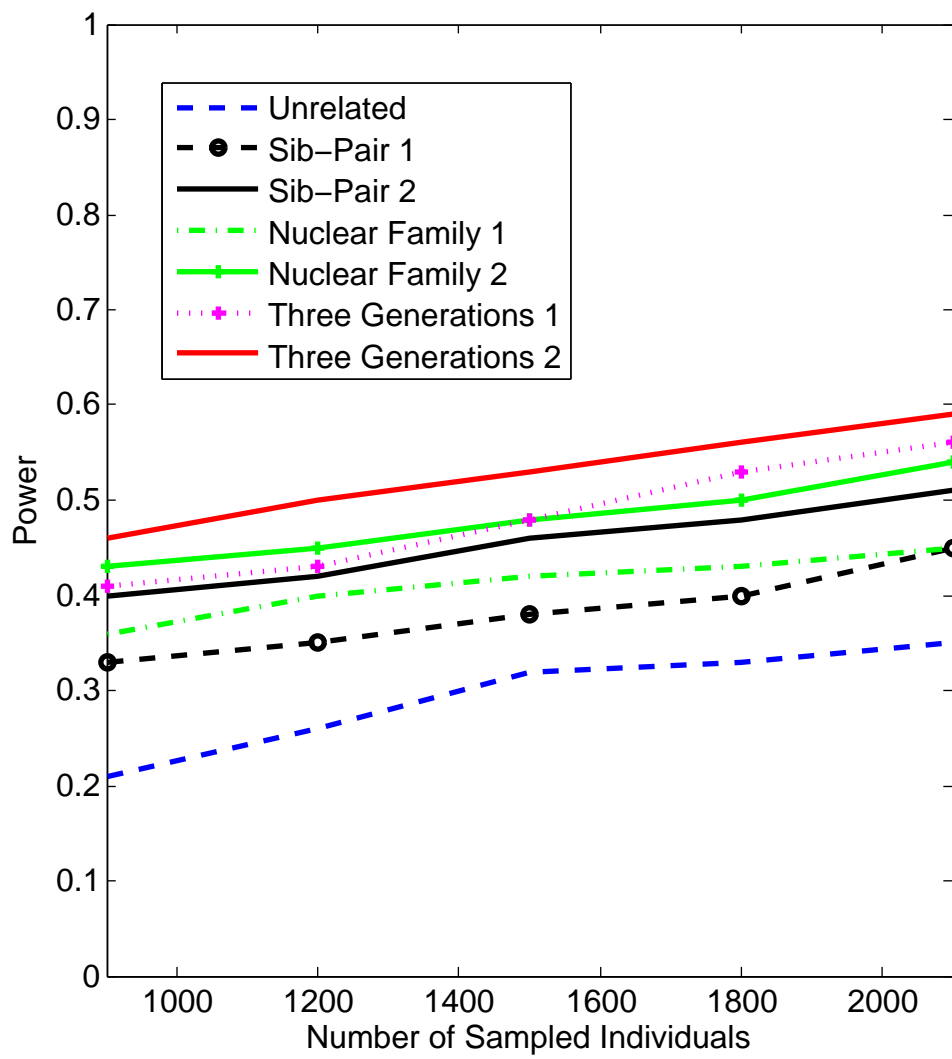

Supplement: Additional file 11: Figure S3C — The power curves of the family-based VT test statistic as a function of the total number of individuals at the significance level α = 0.05 in the test under seven settings: unrelated individuals in cases-controls study, nuclear family groups 1 and 2, sib-pair groups 1 and 2 and three generation family groups 1 and 2, assuming a recessive model, 20% of the risk variants and a baseline penetrance of 0.01. [file 1471-2164-13-667-S11.pdf]

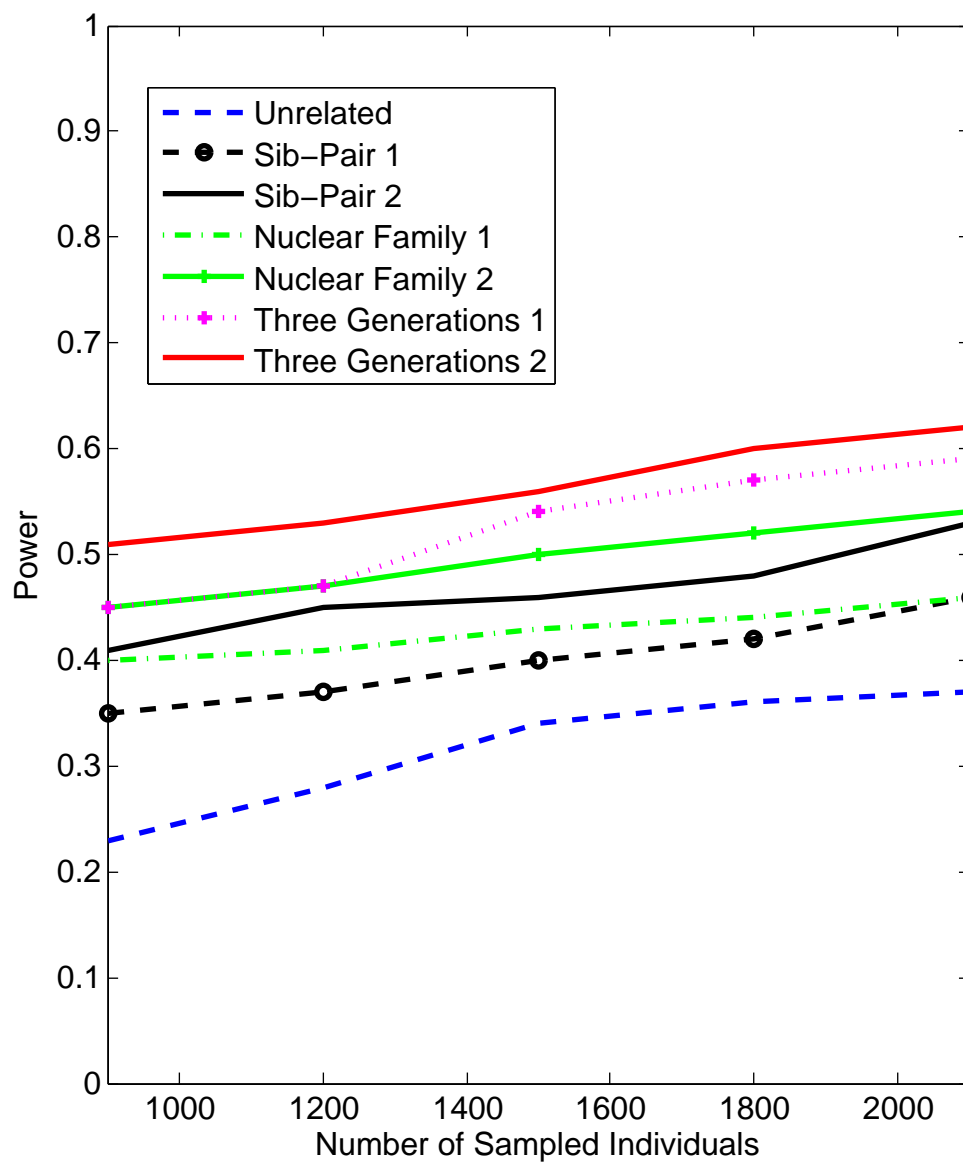

Supplement: Additional file 12: Figure S3D — The power curves of the family-based WSS test statistic as a function of the total number of individuals at the significance level α = 0.05 in the test under seven settings: unrelated individuals in cases-controls study, nuclear family groups 1 and 2, sib-pair groups 1 and 2 and three generation family groups 1 and 2, assuming a recessive model, 20% of the risk variants and a baseline penetrance of 0.01. [file 1471-2164-13-667-S12.pdf]

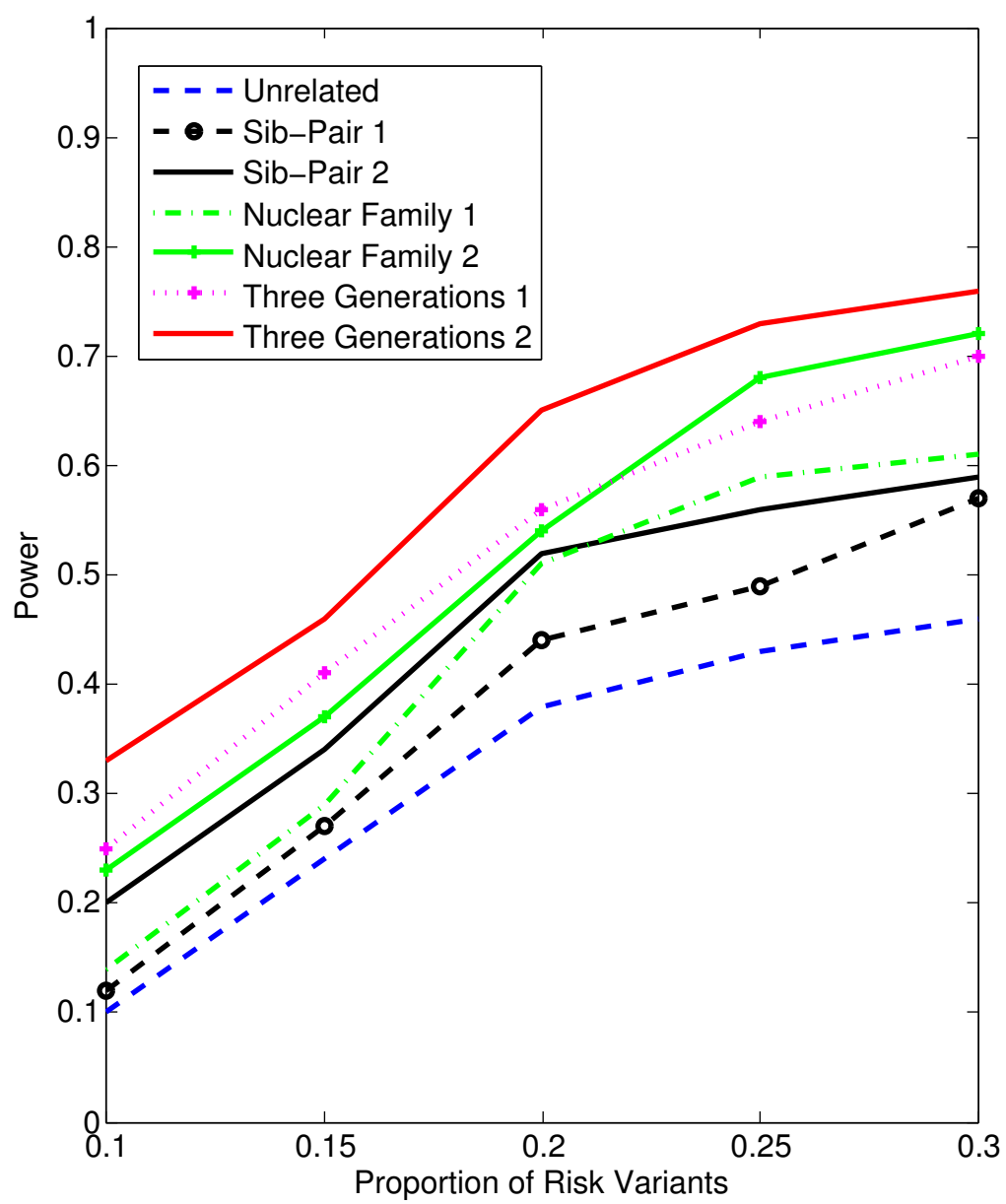

Figure 13

Supplement: Additional file 13: Figure 4A — The power curves of the family-based corrected single marker χ2 test statistic as a function of the proportion of risk variants at the significance level α = 0.05 in the test under seven settings: unrelated individuals in cases-controls study, nuclear family groups 1 and 2, sib-pair groups 1 and 2 and three generation family groups 1 and 2, assuming an additive model, a total of 1,800 sampled individuals and a baseline penetrance of 0.01. [file 1471-2164-13-667-S13.pdf]

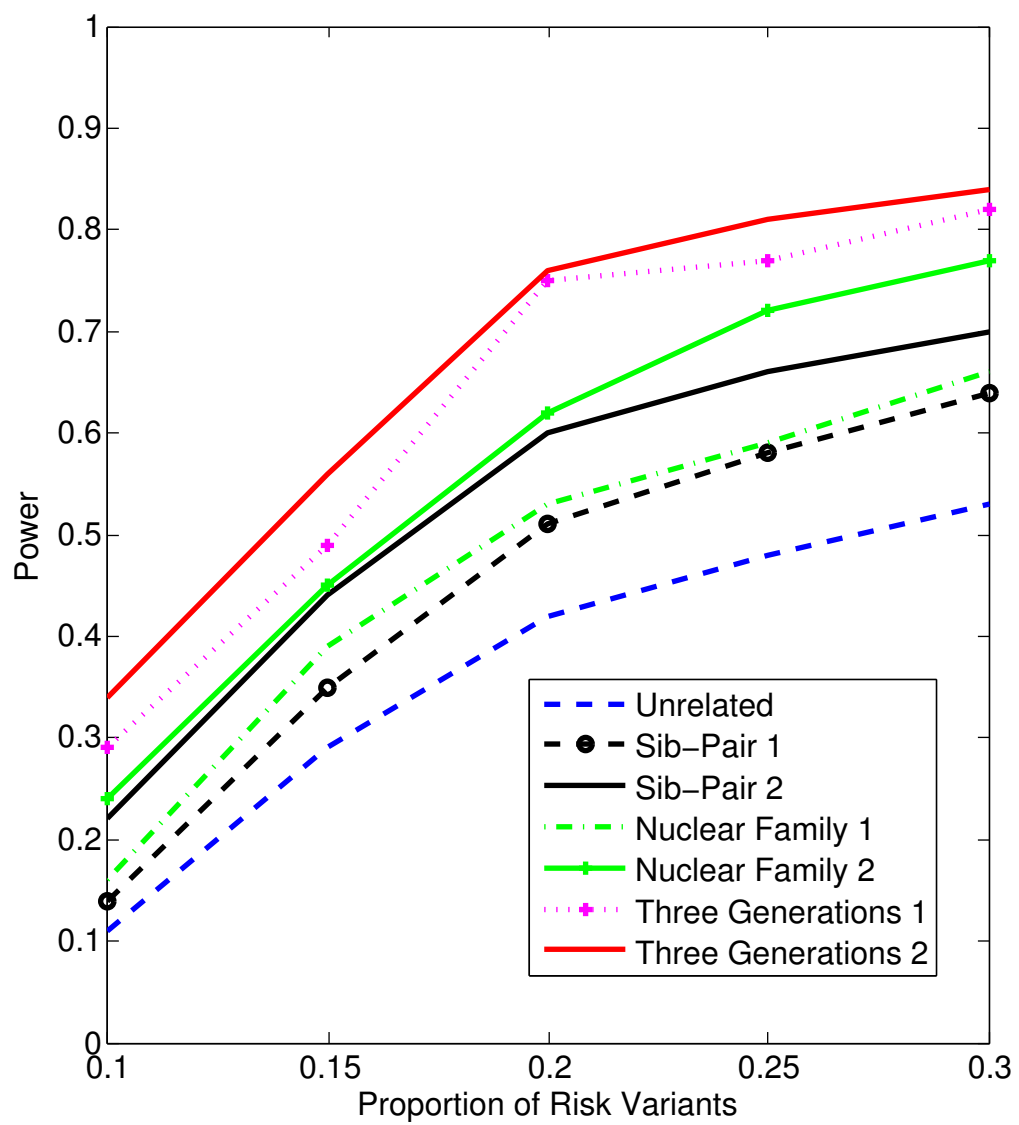

Figure 14

Supplement: Additional file 14: Figure 4B — The power curves of the family-based collapsing test (variants with frequencies ≤0.005 were collapsed) statistic as a function of the proportion of risk variants at the significance level α = 0.05 in the test under seven settings: unrelated individuals in cases-controls study, nuclear family groups 1 and 2, sib-pair groups 1 and 2 and three generation family groups 1 and 2, assuming an additive model, a total of 1,800 sampled individuals and a baseline penetrance of 0.01. [file 1471-2164-13-667-S14.pdf]

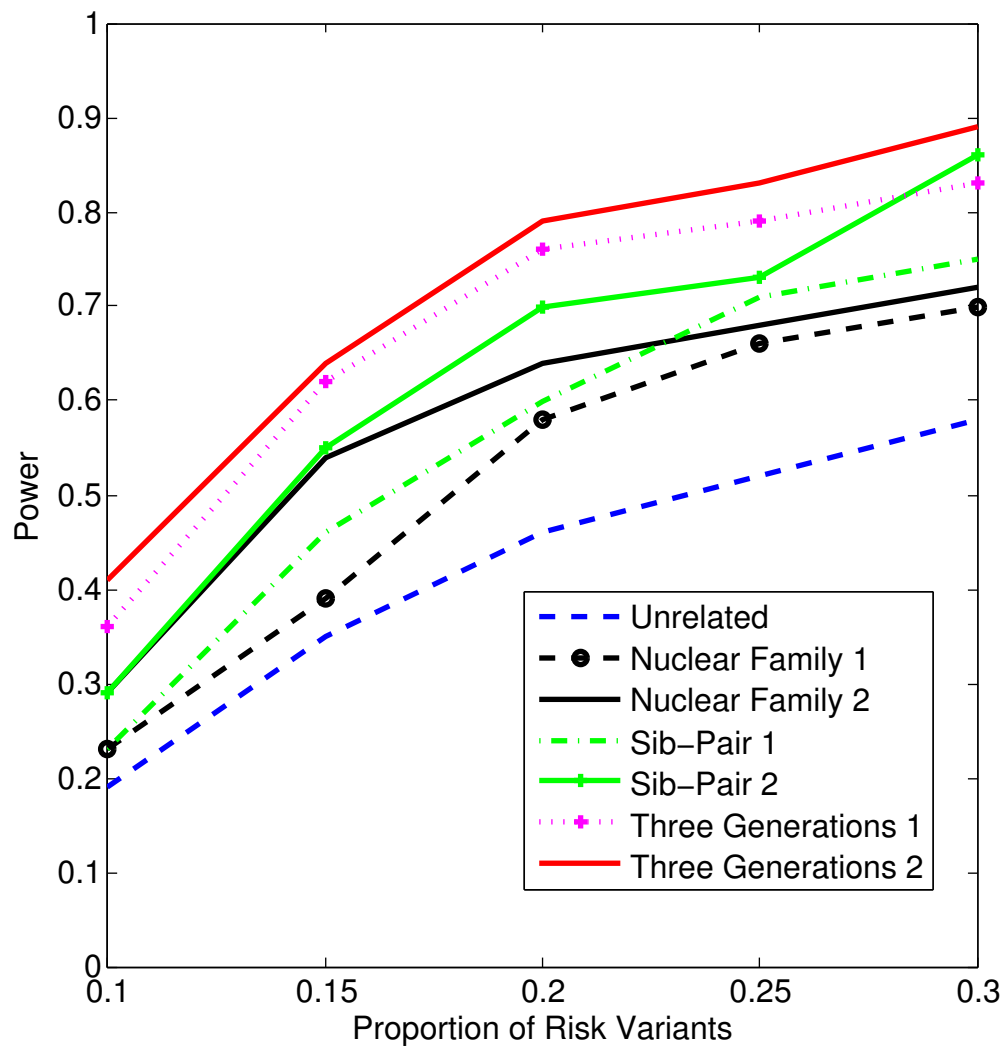

Figure 15

Supplement: Additional file 15: Figure 4C — The power curves of the family-based VT test statistic as a function of the proportion of risk variants at the significance level α = 0.05 in the test under seven settings: unrelated individuals in cases-controls study, nuclear family groups 1 and 2, sib-pair groups 1 and 2 and three generation family groups 1 and 2, assuming an additive model, a total of 1,800 sampled individuals and a baseline penetrance of 0.01. [file 1471-2164-13-667-S15.pdf]

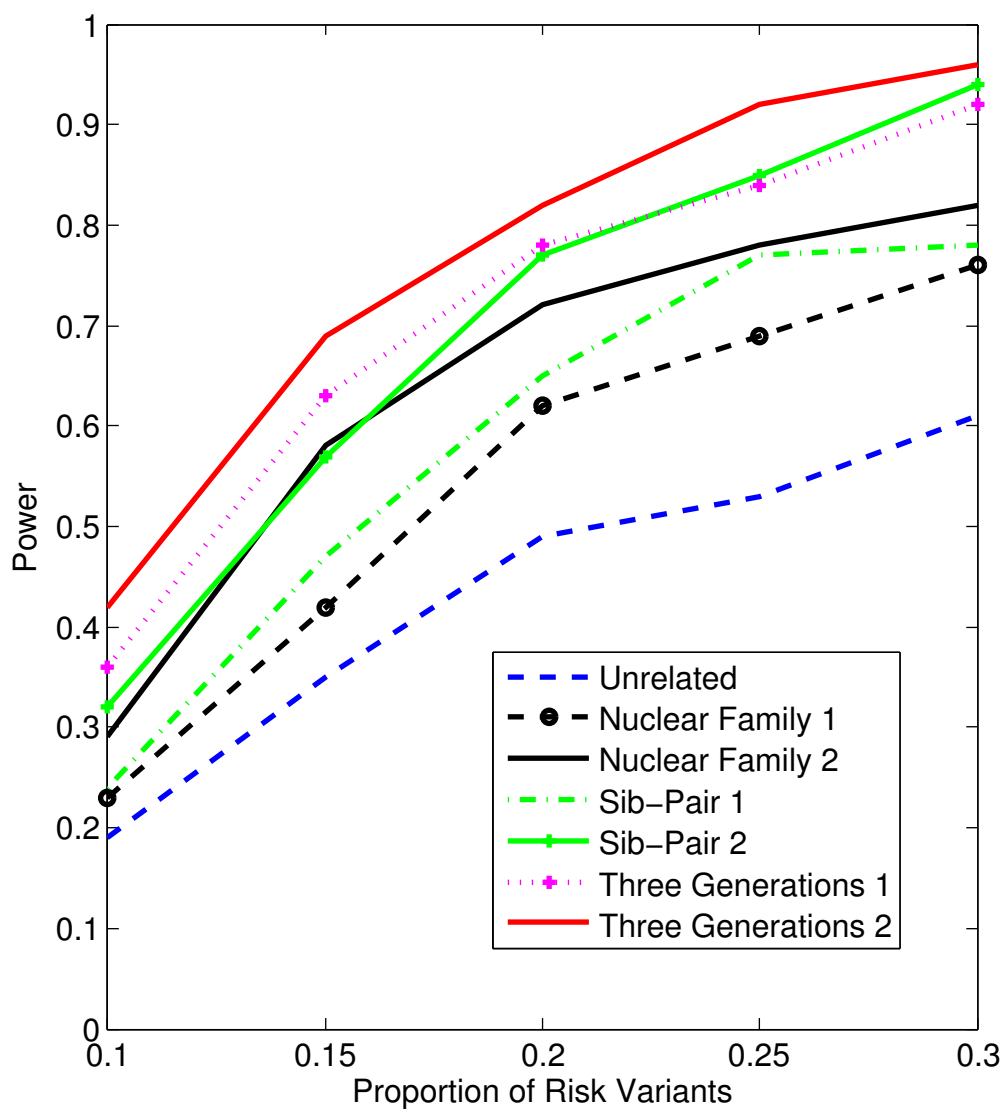

Figure 16

Supplement: Additional file 16: Figure 4D — The power curves of the family-based WSS test statistic as a function of the proportion of risk variants at the significance level α = 0.05 in the test under seven settings: unrelated individuals in cases-controls study, nuclear family groups 1 and 2, sib-pair groups 1 and 2 and three generation family groups 1 and 2, assuming an additive model, a total of 1,800 sampled individuals and a baseline penetrance of 0.01. [file 1471-2164-13-667-S16.pdf]

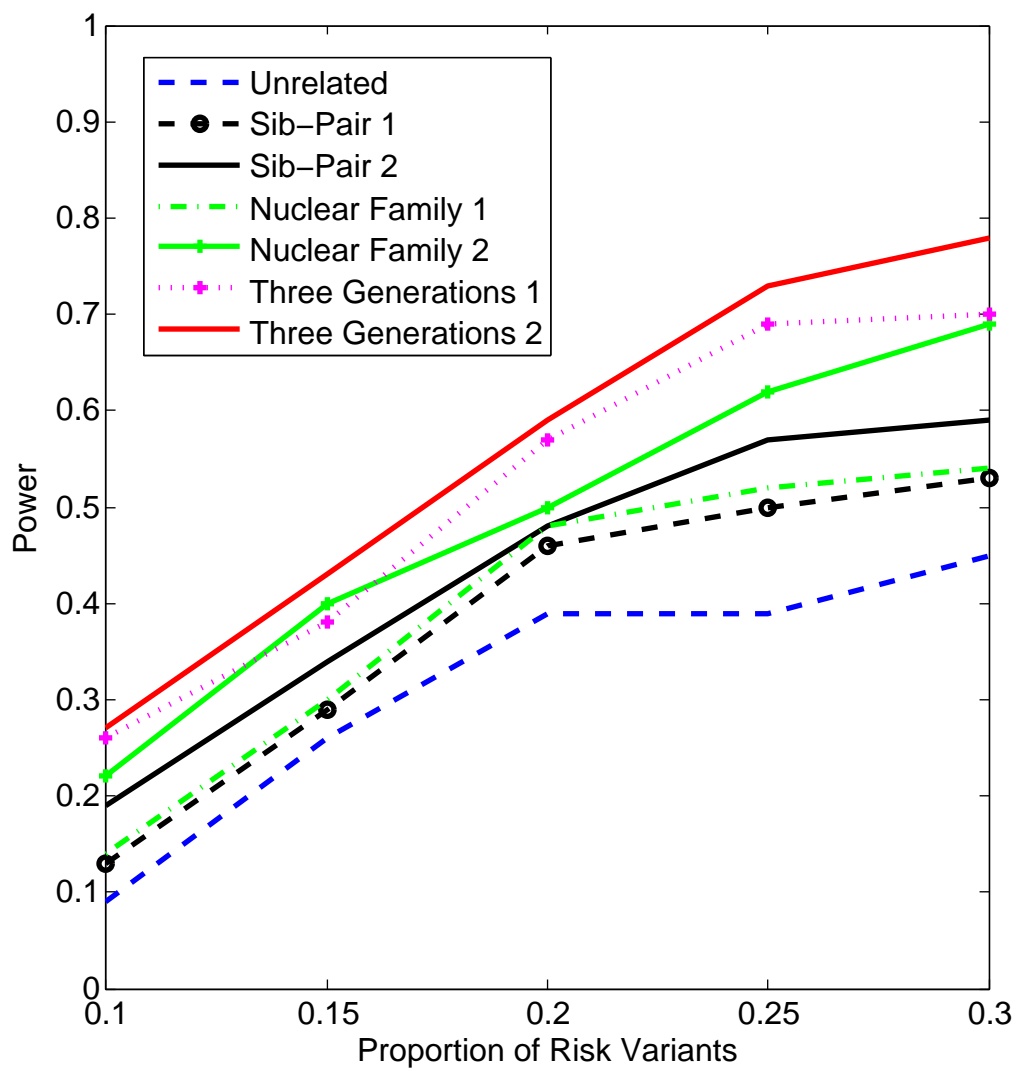

Supplement: Additional file 17: Figure S5A — The power curves of the family-based corrected single marker χ2 test statistic as a function of the proportion of risk variants at the significance level α = 0.05 in the test under seven settings: unrelated individuals in cases-controls study, nuclear family groups 1 and 2, sib-pair groups 1 and 2 and three generation family groups 1 and 2, assuming a multiplicative model, a total of 1,800 sampled individuals and a baseline penetrance of 0.01. [file 1471-2164-13-667-S17.pdf]

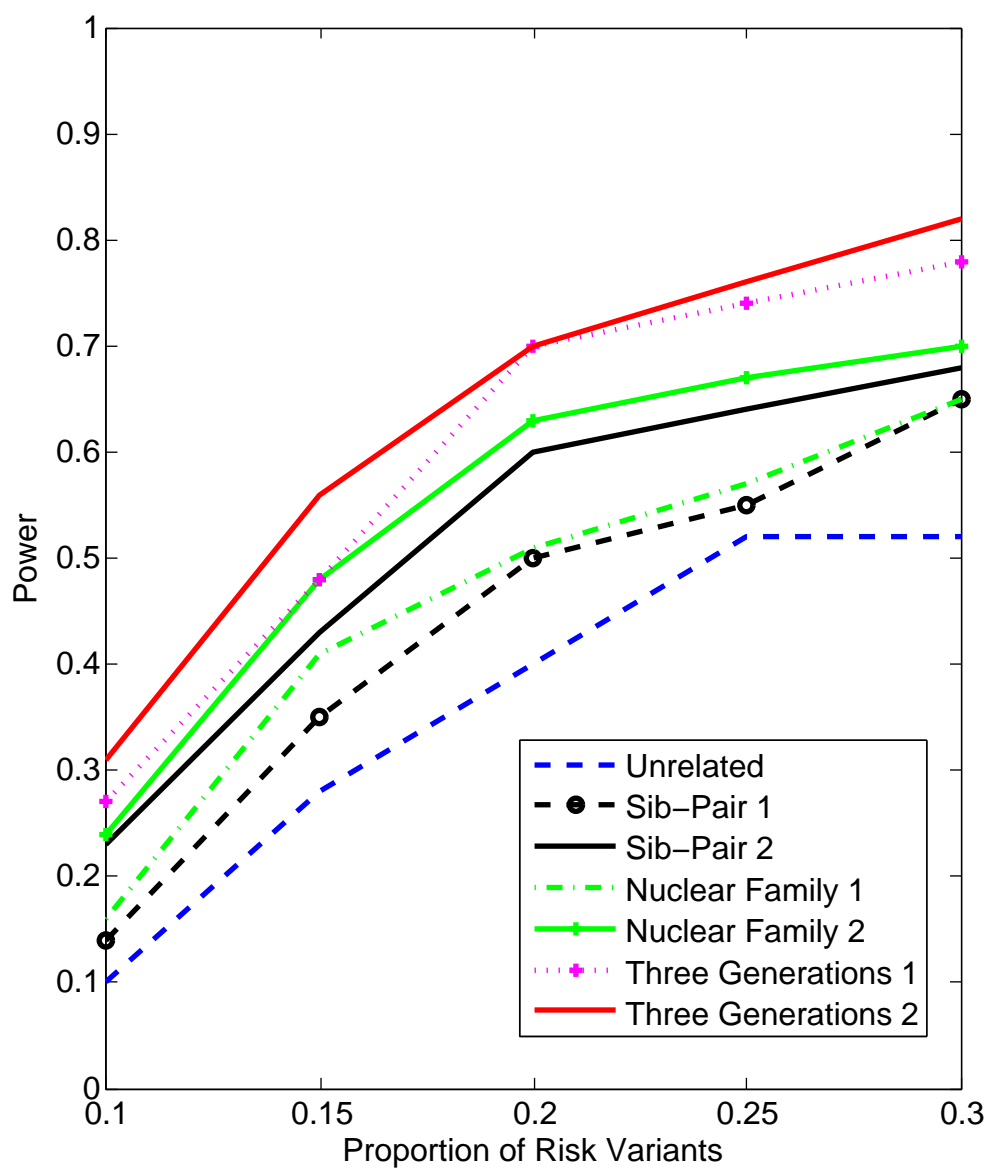

Supplement: Additional file 18: Figure S5B — The power curves of the family-based collapsing test (variants with frequencies ≤0.005 were collapsed) statistic as a function of the proportion of risk variants at the significance level α = 0.05 in the test under seven settings: unrelated individuals in cases-controls study, nuclear family groups 1 and 2, sib-pair groups 1 and 2 and three generation family groups 1 and 2, assuming a multiplicative model, a total of 1,800 sampled individuals and a baseline penetrance of 0.01. [file 1471-2164-13-667-S18.pdf]

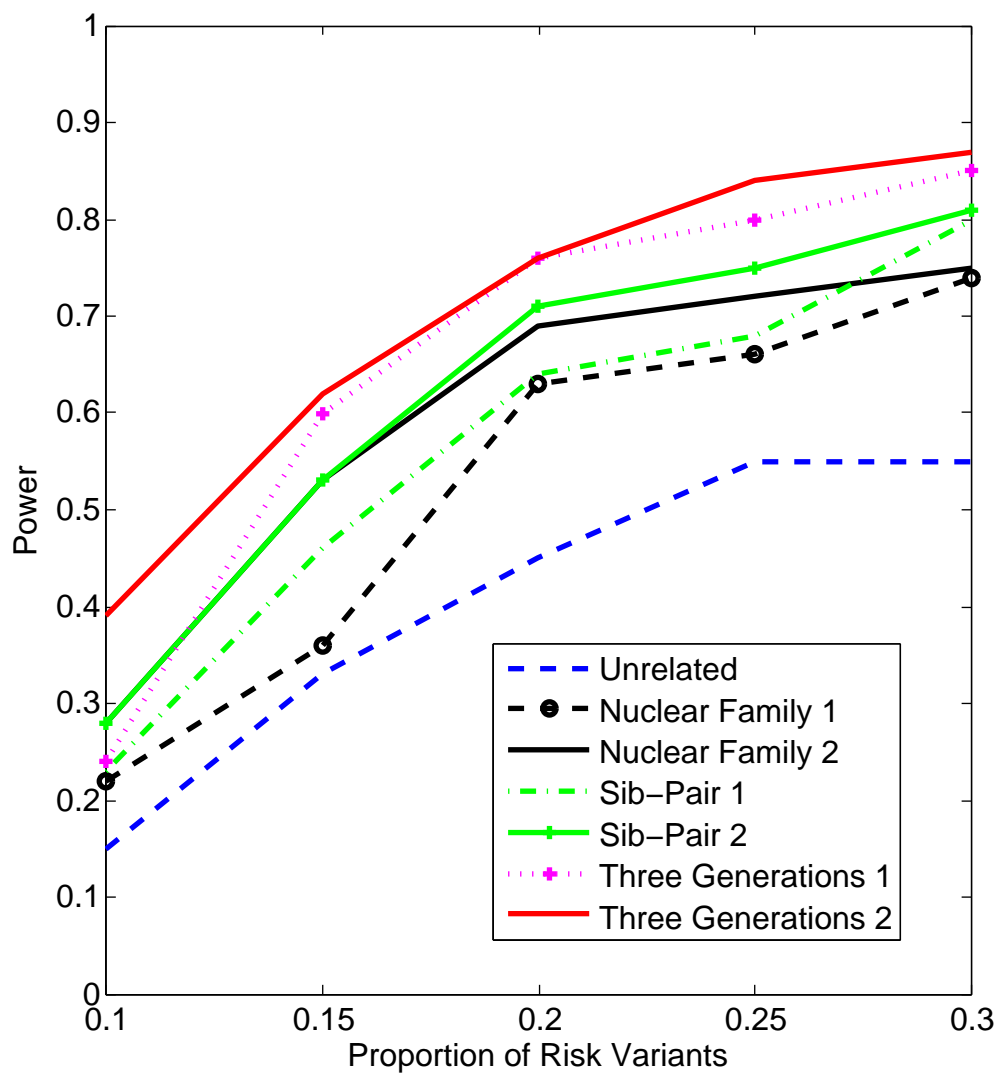

Supplement: Additional file 19: Figure S5C — The power curves of the family-based VT test statistic as a function of the proportion of risk variants at the significance level α = 0.05 in the test under seven settings: unrelated individuals in cases-controls study, nuclear family groups 1 and 2, sib-pair groups 1 and 2 and three generation family groups 1 and 2, assuming the multiplicative model, a total of 1,800 sampled individuals and a baseline penetrance of 0.01. [file 1471-2164-13-667-S19.pdf]

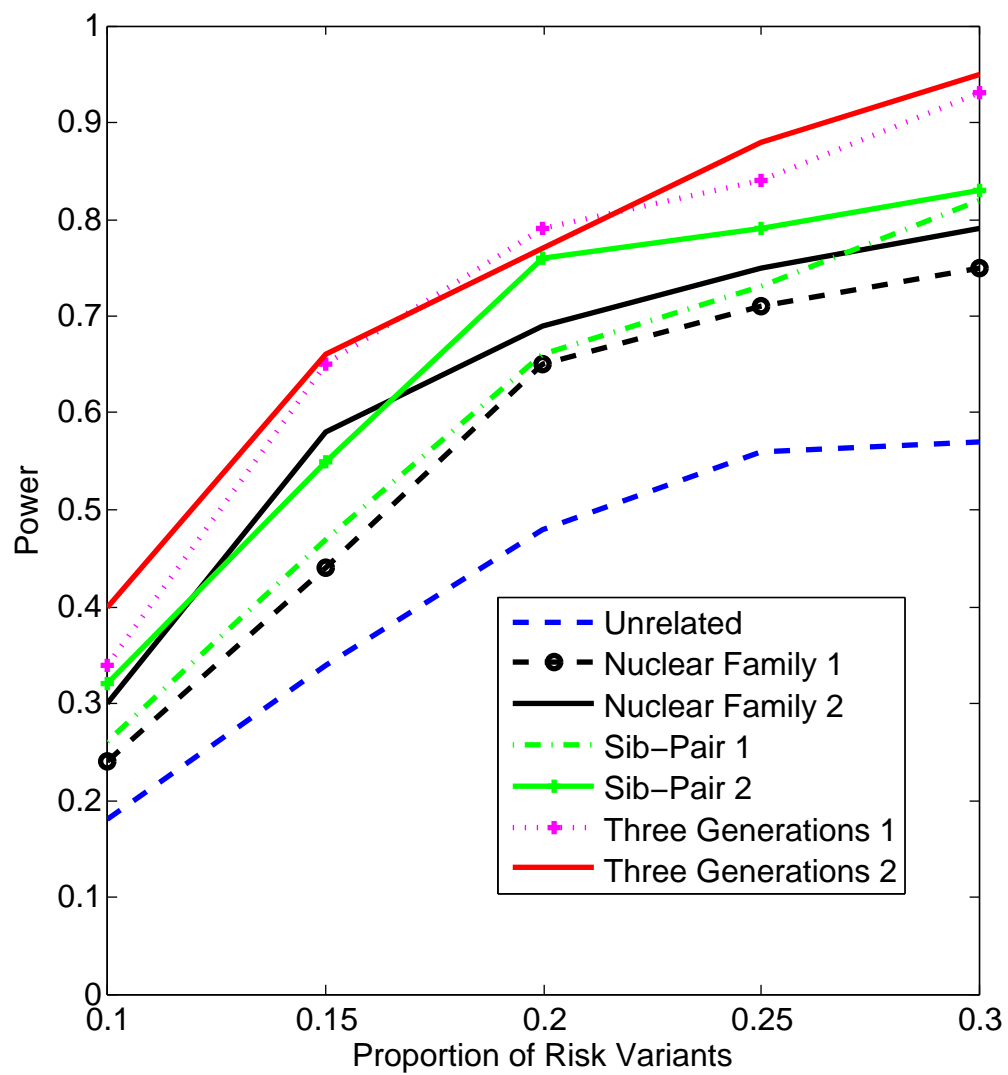

Supplement: Additional file 20: Figure S5D — The power curves of the family-based WSS test statistic as a function of the proportion of risk variants at the significance level α = 0.05 in the test under seven settings: unrelated individuals in cases-controls study, nuclear family groups 1 and 2, sib-pair groups 1 and 2 and three generation family groups 1 and 2, assuming the multiplicative model, a total of 1,800 sampled individuals and a baseline penetrance of 0.01. [file 1471-2164-13-667-S20.pdf]

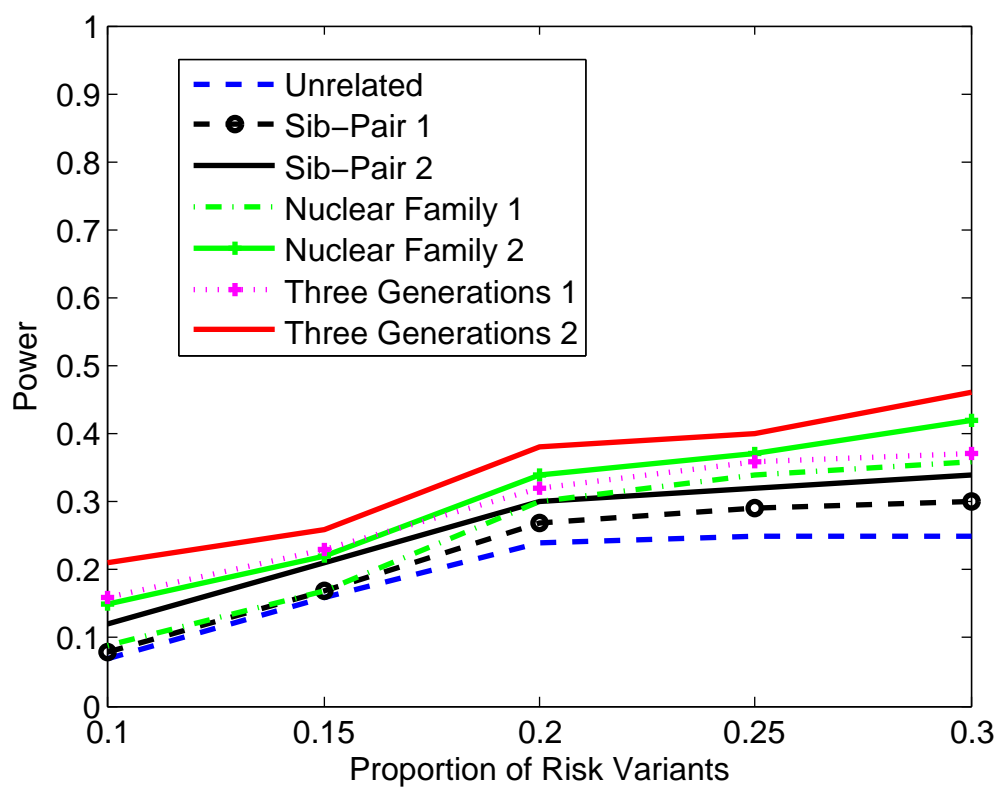

Supplement: Additional file 21: Figure S6A — The power curves of the family-based corrected single marker χ2 test statistic as a function of the proportion of risk variants at the significance level α = 0.05 in the test under seven settings: unrelated individuals in cases-controls study, nuclear family groups 1 and 2, sib-pair groups 1 and 2 and three generation family groups 1 and 2, assuming a recessive model, a total of 1,800 sampled individuals and a baseline penetrance of 0.01. [file 1471-2164-13-667-S21.pdf]

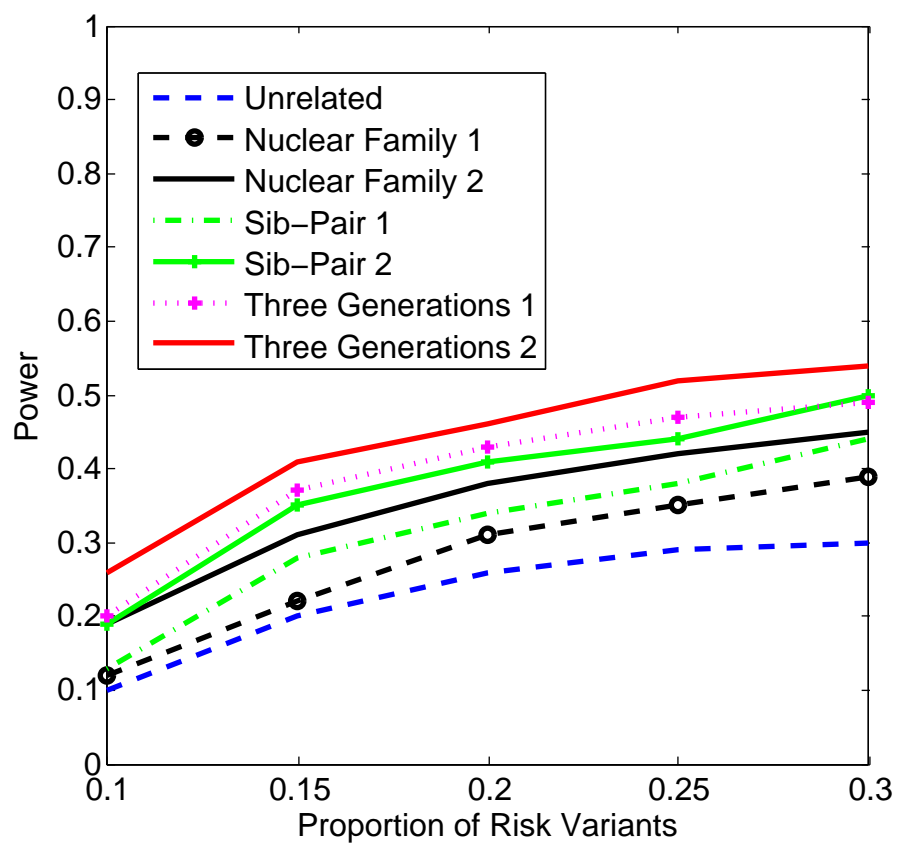

Supplement: Additional file 23: Figure S6C — The power curves of the family-based VT test statistic as a function of the proportion of risk variants at the significance level α = 0.05 in the test under seven settings: unrelated individuals in cases-controls study, nuclear family groups 1 and 2, sib-pair groups 1 and 2 and three generation family groups 1 and 2, assuming the recessive model, a total of 1,800 sampled individuals and a baseline penetrance of 0.01. [file 1471-2164-13-667-S23.pdf]

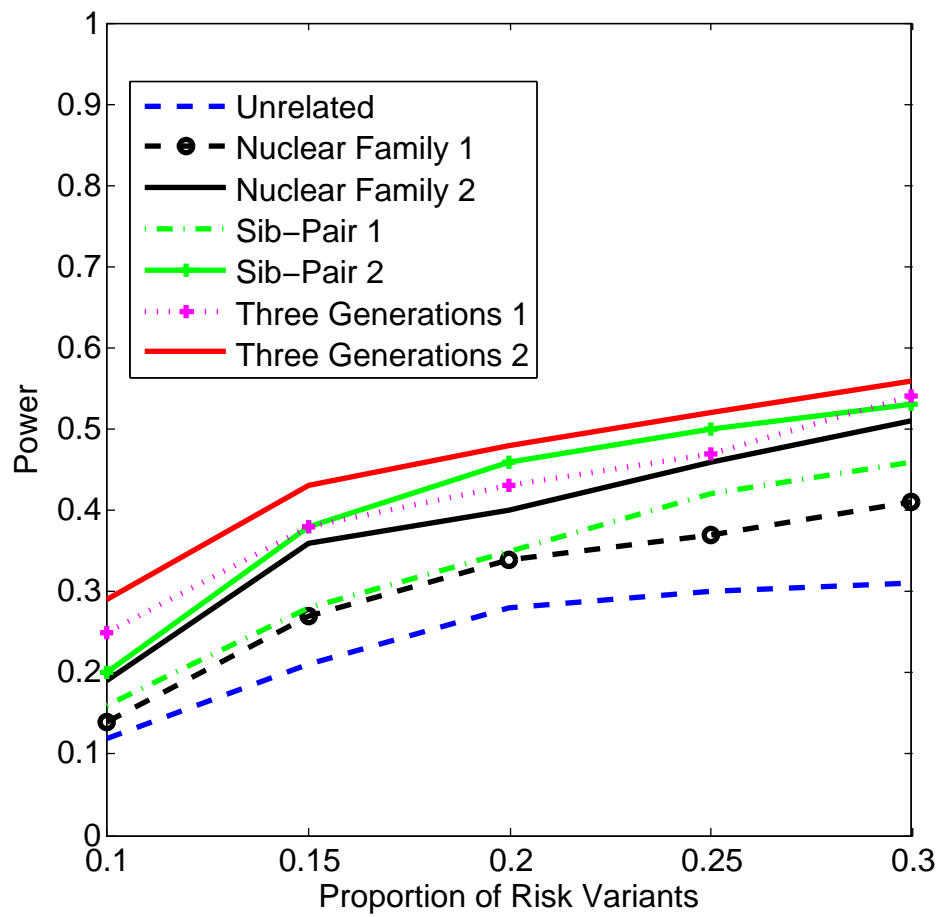

Supplement: Additional file 24: Figure S6D — The power curves of the family-based WSS test statistic as a function of the proportion of risk variants at the significance level α = 0.05 in the test under seven settings: unrelated individuals in cases-controls study, nuclear family groups 1 and 2, sib-pair groups 1 and 2 and three generation family groups 1 and 2, assuming the recessive model, a total of 1,800 sampled individuals and a baseline penetrance of 0.01. [file 1471-2164-13-667-S24.pdf]

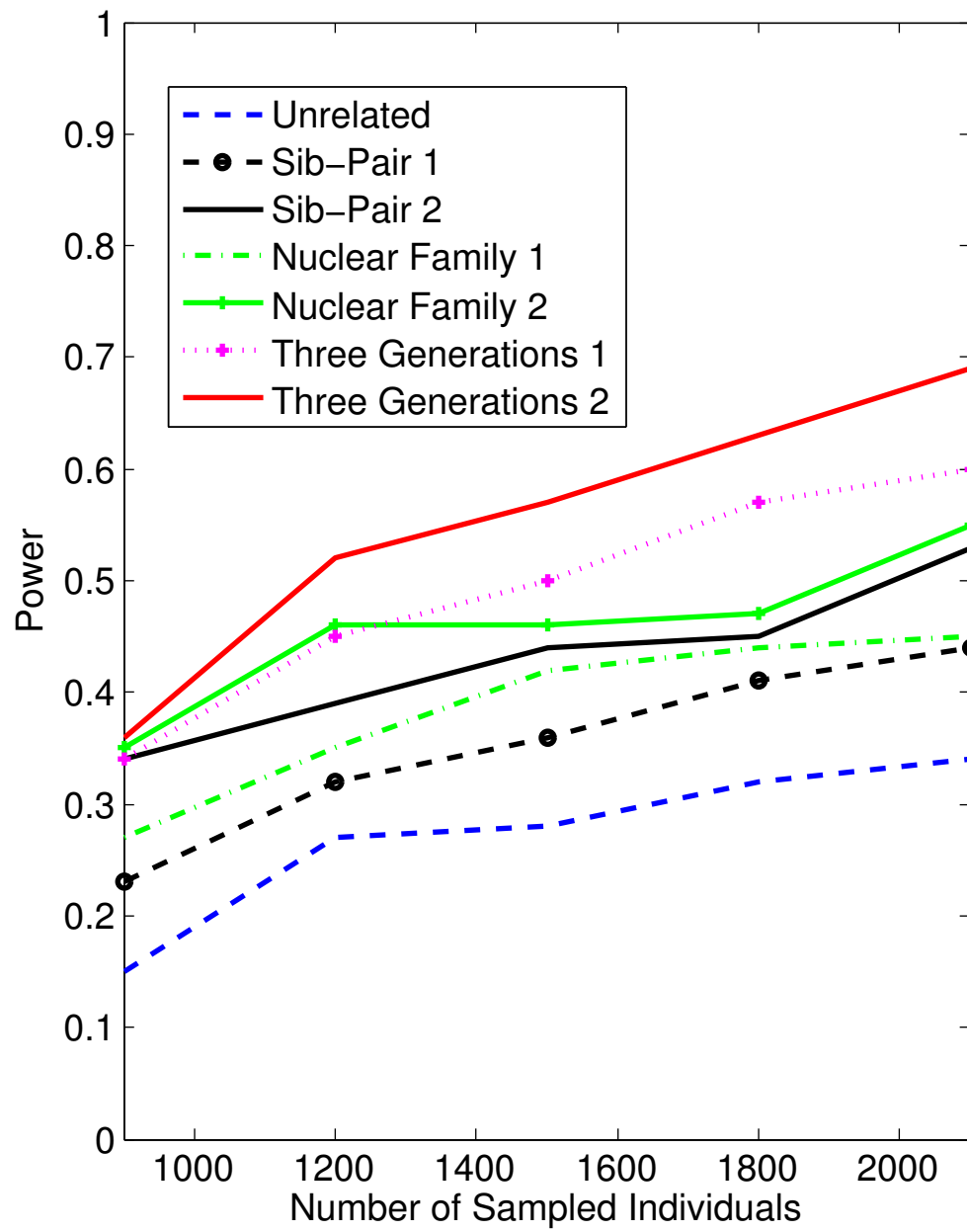

Figure 21

Supplement: Additional file 25: Figure S7A — The power curves of the family-based corrected single marker χ2 statistic under opposite directions of association as a function of the total number of individuals at the significance level α = 0.05 in the test under seven settings: unrelated individuals in cases-controls study, nuclear family groups 1 and 2, sib-pair groups 1 and 2 and three generation family groups 1 and 2, assuming an additive model, 20% of the risk variants and a baseline penetrance of 0.01. [file 1471-2164-13-667-S25.pdf]

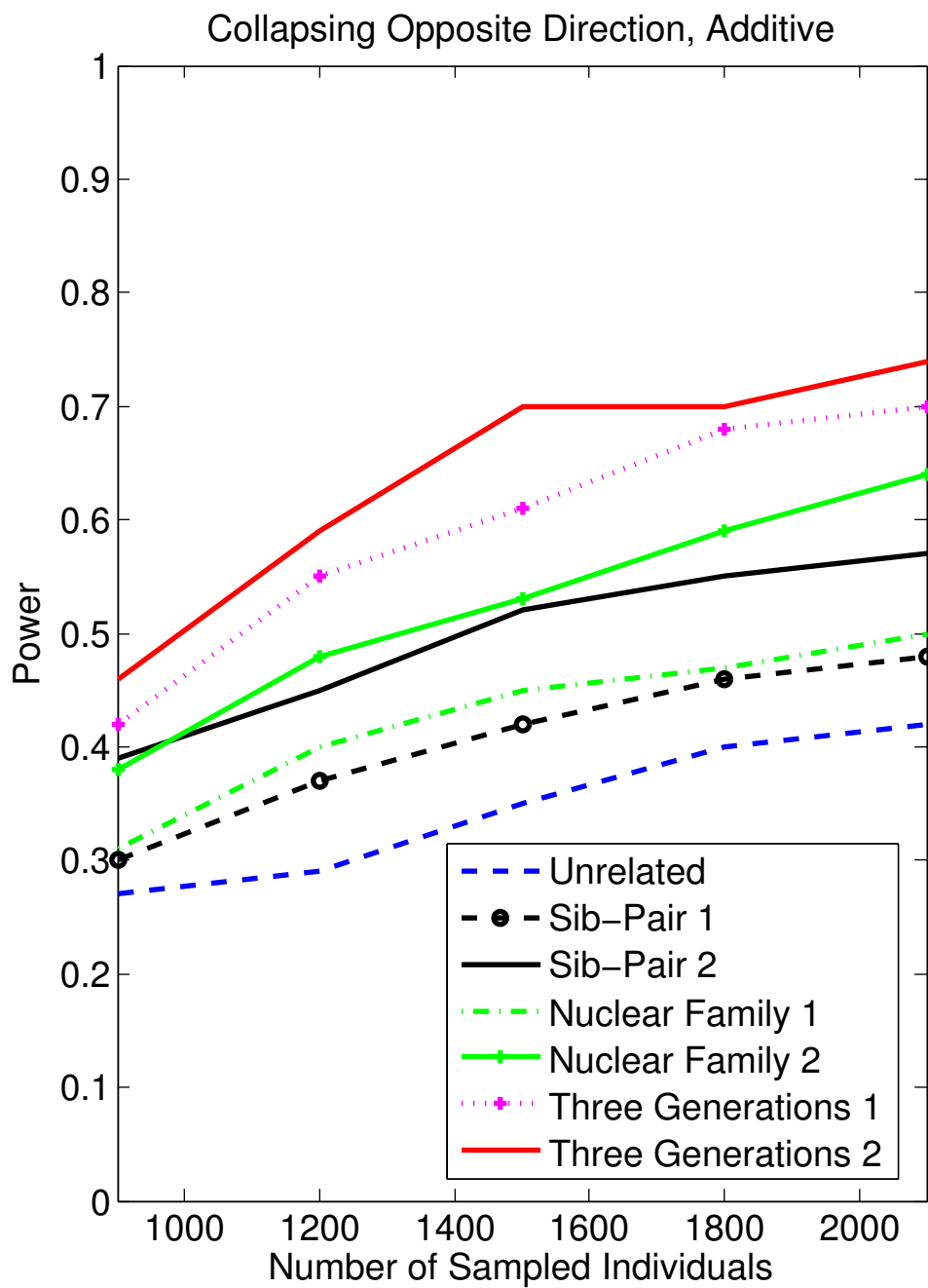

Figure 22

Supplement: Additional file 26: Figure S7B — The power curves of the family-based collapsing test (variants with frequencies ≤0.005 were collapsed) statistic under opposite directions of association as a function of the total number of individuals at the significance level α = 0.05 in the test under seven settings: unrelated individuals in cases-controls study, nuclear family groups 1 and 2, sib-pair groups 1 and 2 and three generation family groups 1 and 2, assuming an additive model, 20% of the risk variants and a baseline penetrance of 0.01. [file 1471-2164-13-667-S26.pdf]

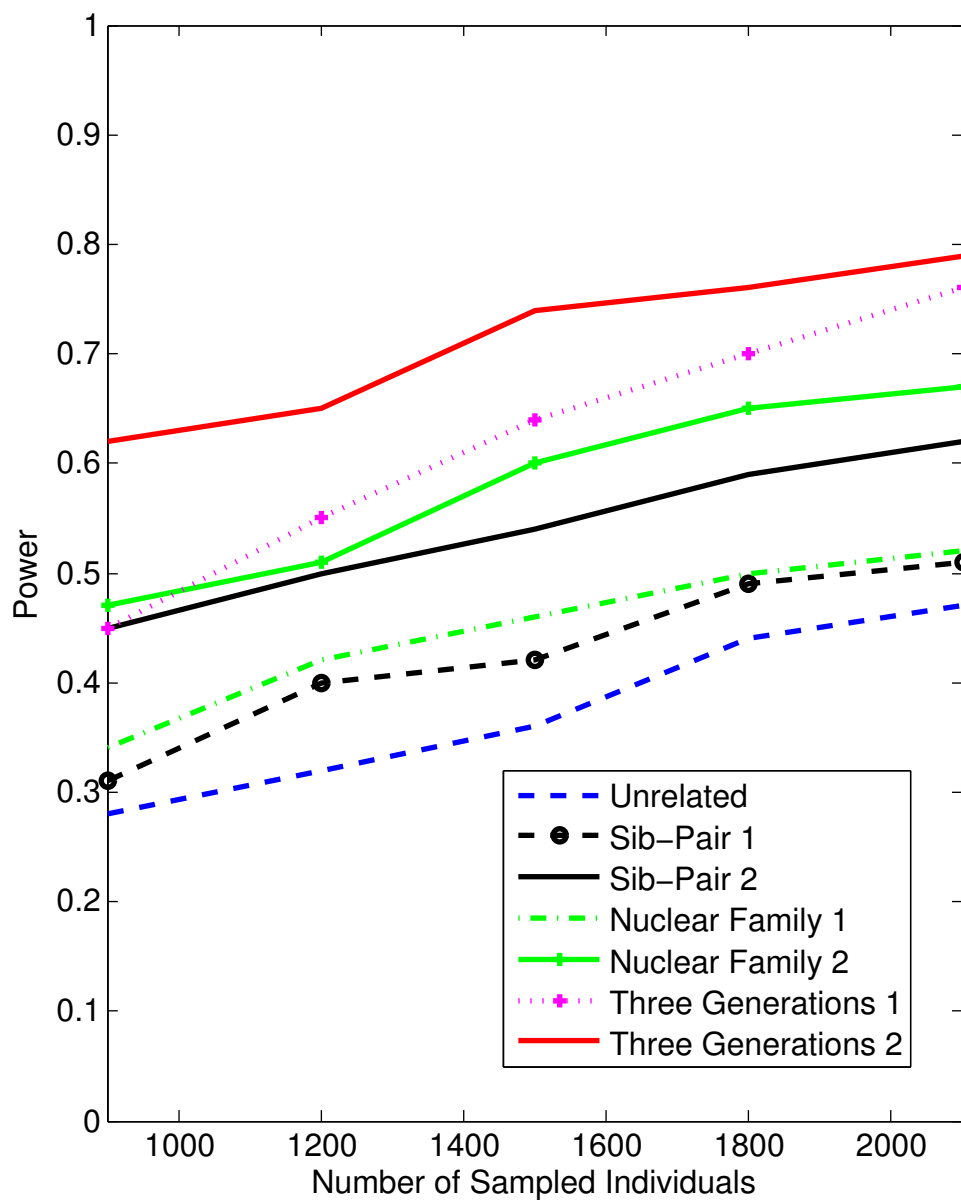

Figure 23

Supplement: Additional file 27: Figure S7C — The power curves of the family-based VT statistic under opposite directions of association as a function of the total number of individuals at the significance level α = 0.05 in the test under seven settings: unrelated individuals in cases-controls study, nuclear family groups 1 and 2, sib-pair groups 1 and 2 and three generation family groups 1 and 2, assuming an additive model, 20% of the risk variants and a baseline penetrance of 0.01. [file 1471-2164-13-667-S27.pdf]

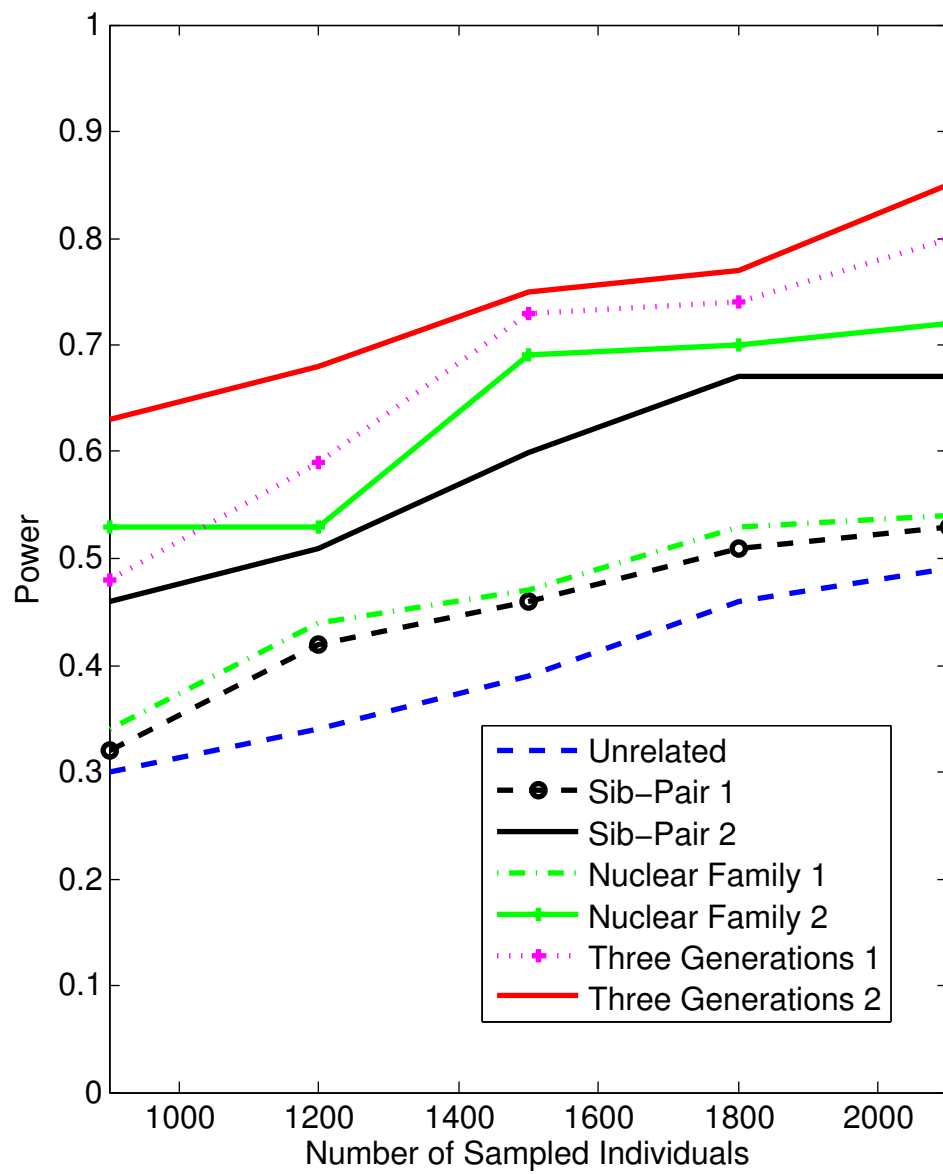

Figure 24

Supplement: Additional file 28: Figure S7D — The power curves of the family-based WSS test statistic under opposite directions of association as a function of the total number of individuals at the significance level α = 0.05 in the test under seven settings: unrelated individuals in cases-controls study, nuclear family groups 1 and 2, sib-pair groups 1 and 2 and three generation family groups 1 and 2, assuming an additive model, 20% of the risk variants and a baseline penetrance of 0.01. [file 1471-2164-13-667-S28.pdf]

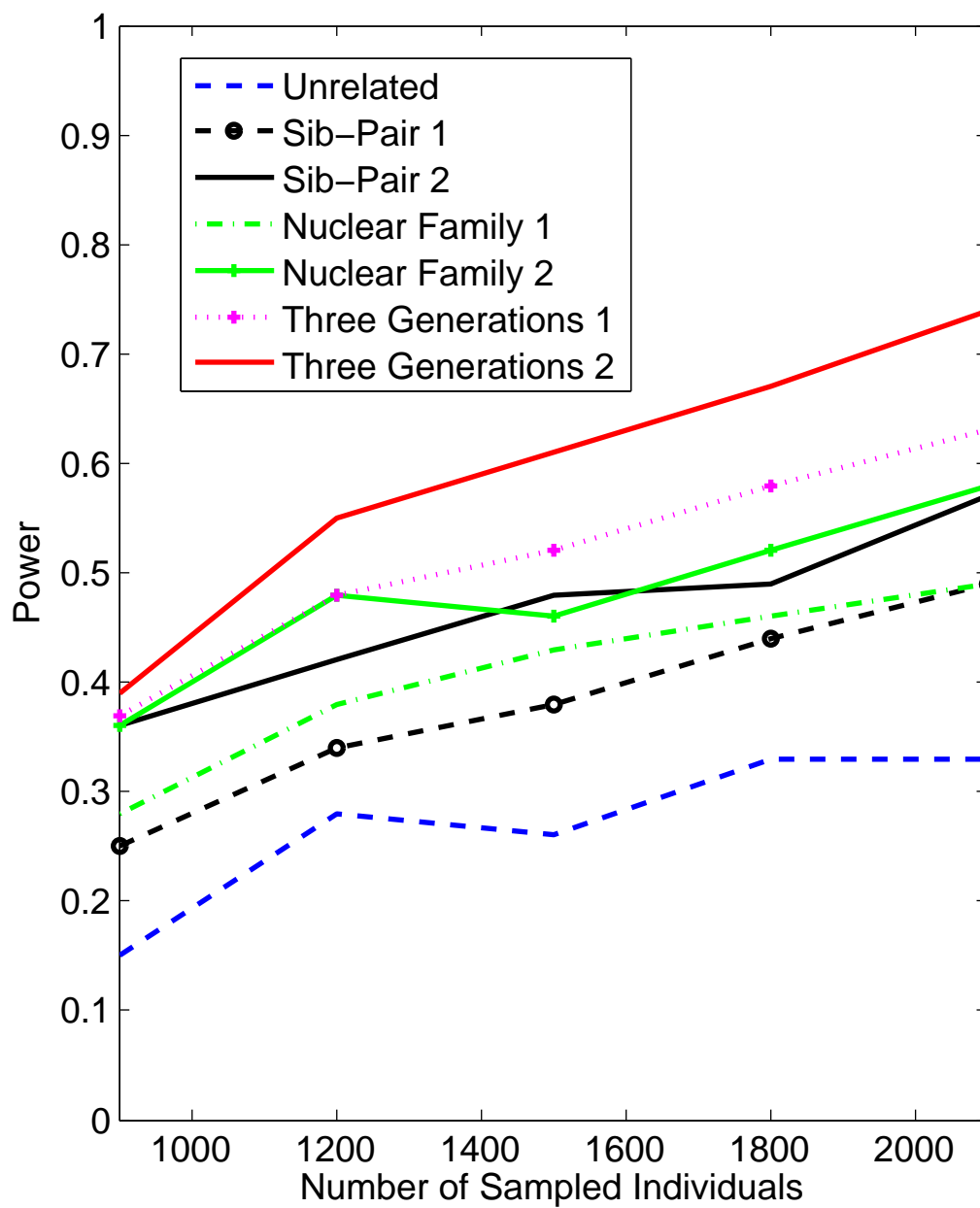

Supplement: Additional file 29: Figure S8A — The power curves of the family-based corrected single marker χ2 statistic under opposite directions of association as a function of the total number of individuals at the significance level α = 0.05 in the test under seven settings: unrelated individuals in cases-controls study, nuclear family groups 1 and 2, sib-pair groups 1 and 2 and three generation family groups 1 and 2, assuming a multiplicative model, 20% of the risk variants and a baseline penetrance of 0.01. [file 1471-2164-13-667-S29.pdf]

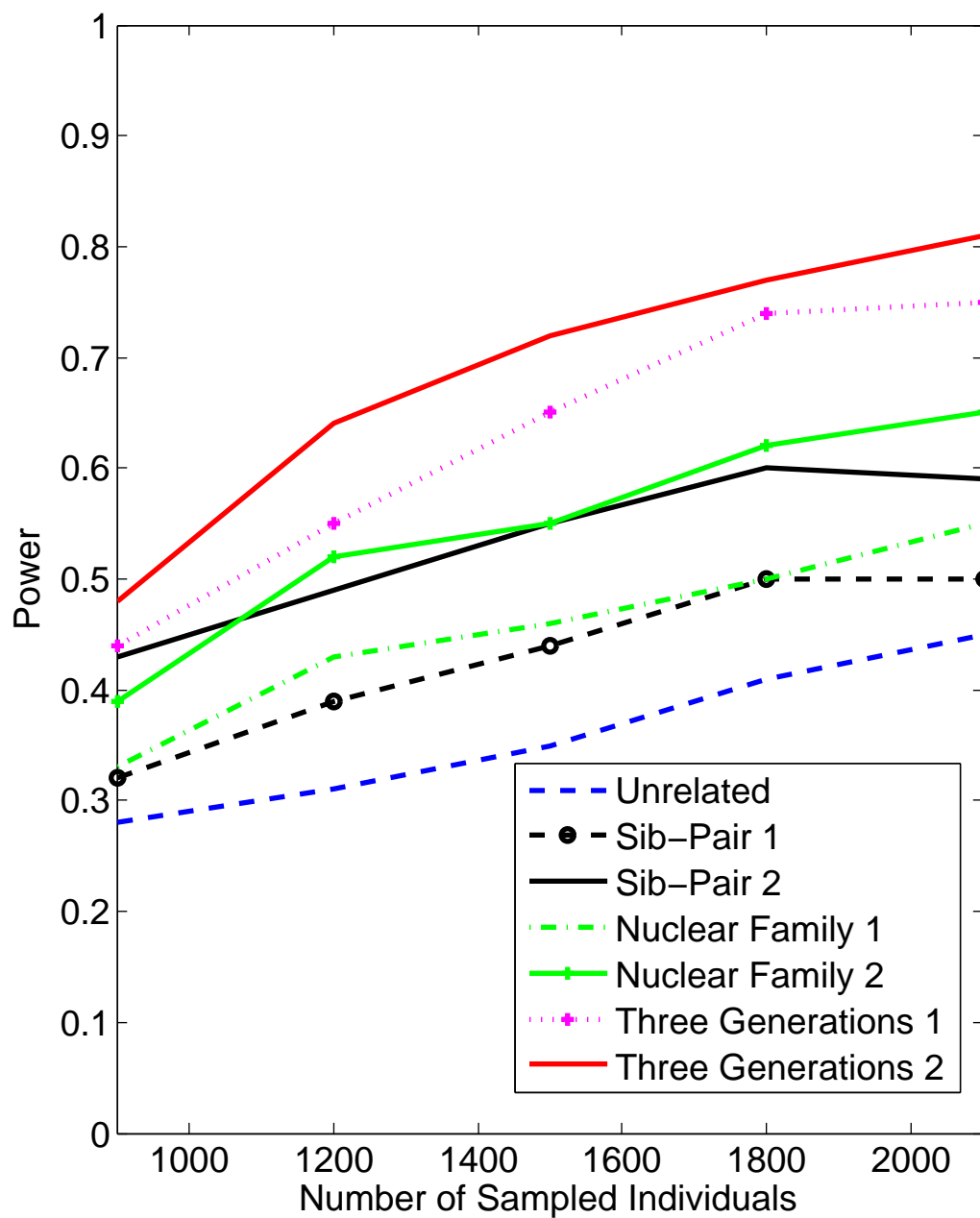

Supplement: Additional file 30: Figure S8B — The power curves of the family-based collapsing test (variants with frequencies ≤0.005 were collapsed) statistic under opposite directions of association as a function of the total number of individuals at the significance level α = 0.05 in the test under seven settings: unrelated individuals in cases-controls study, nuclear family groups 1 and 2, sib-pair groups 1 and 2 and three generation family groups 1 and 2, assuming a multiplicative model, 20% of the risk variants and a baseline penetrance of 0.01. [file 1471-2164-13-667-S30.pdf]

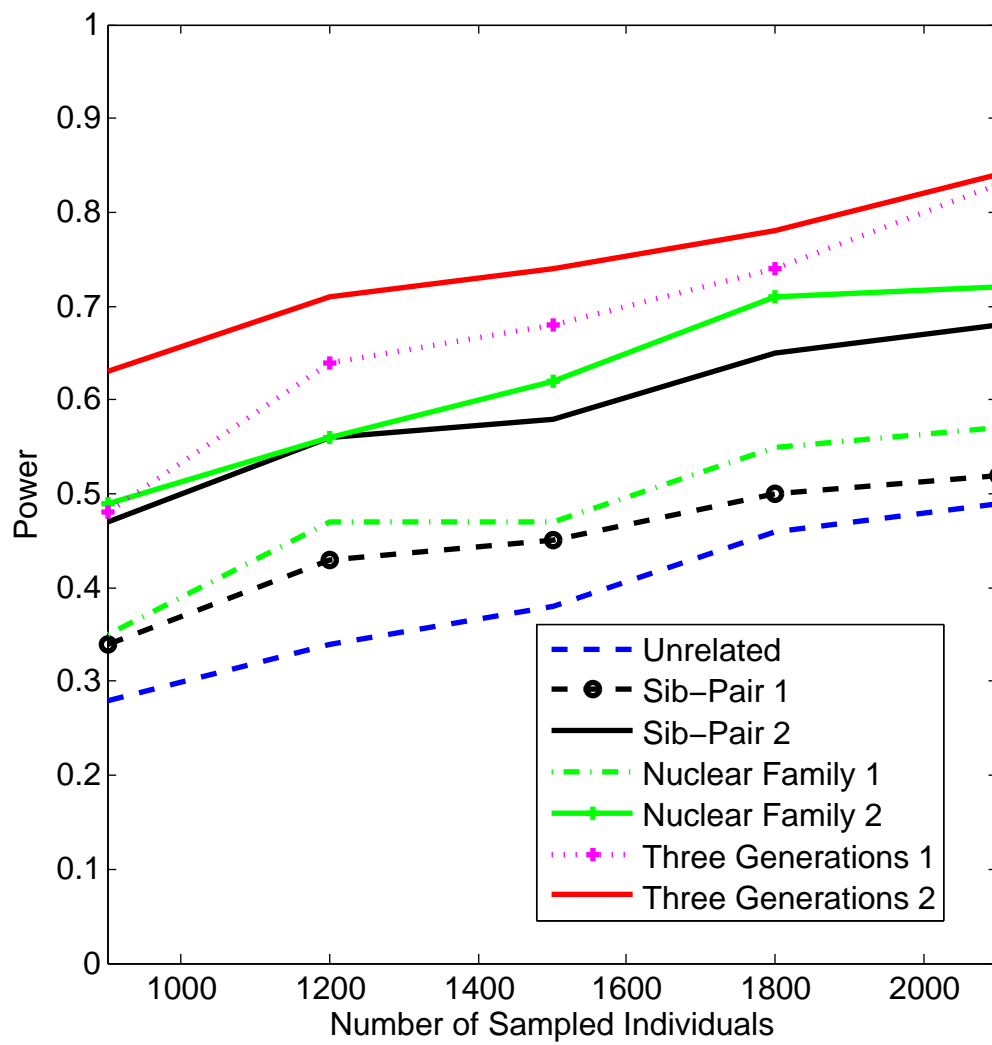

Supplement: Additional file 31: Figure S8C — The power curves of the family-based VT statistic under opposite directions of association as a function of the total number of individuals at the significance level α = 0.05 in the test under seven settings: unrelated individuals in cases-controls study, nuclear family groups 1 and 2, sib-pair groups 1 and 2 and three generation family groups 1 and 2, assuming a multiplicative model, 20% of the risk variants and a baseline penetrance of 0.01. [file 1471-2164-13-667-S31.pdf]

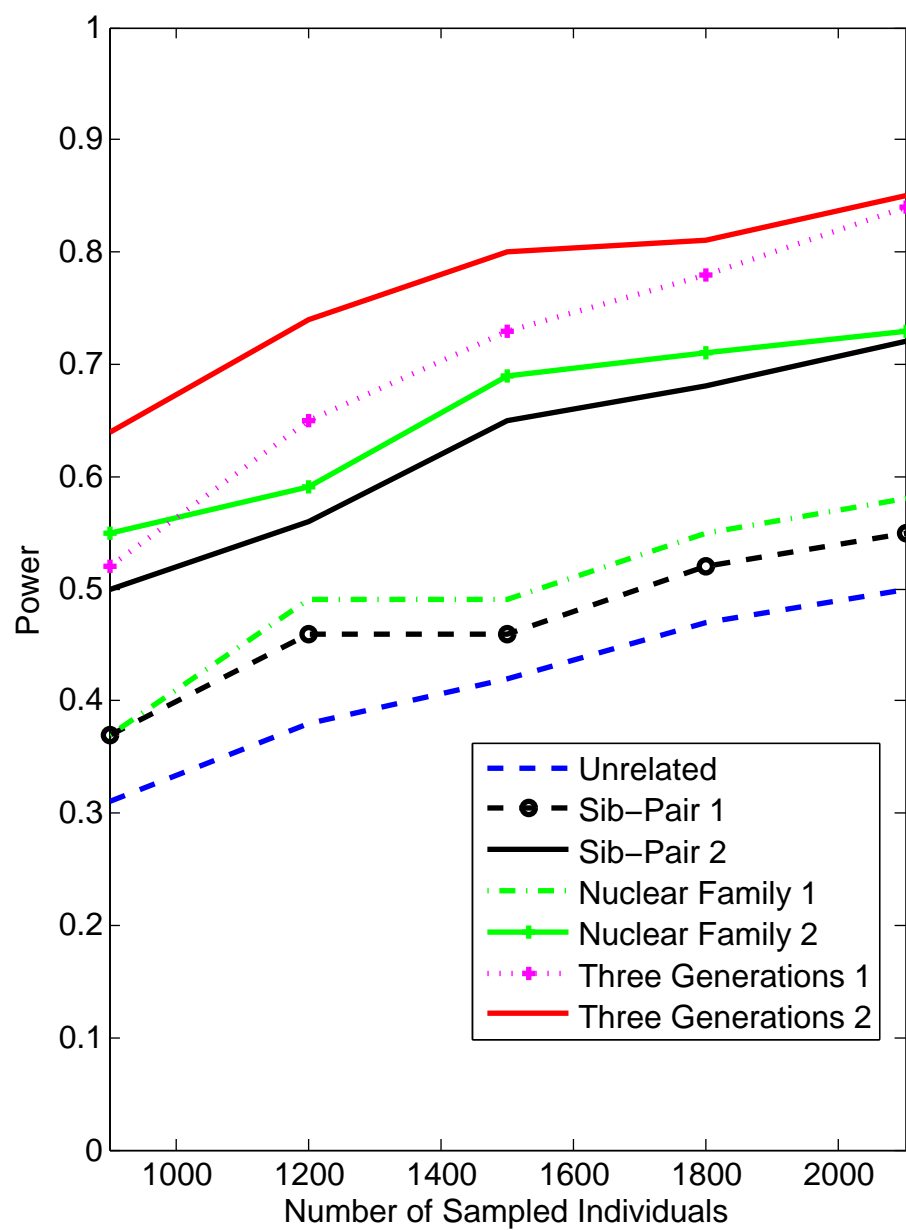

Supplement: Additional file 32: Figure S8D — The power curves of the family-based WSS test statistic under opposite directions of association as a function of the total number of individuals at the significance level α = 0.05 in the test under seven settings: unrelated individuals in cases-controls study, nuclear family groups 1 and 2, sib-pair groups 1 and 2 and three generation family groups 1 and 2, assuming a multiplicative model, 20% of the risk variants and a baseline penetrance of 0.01. (PDF 4 kb) (PDF 4 kb) [file 1471-2164-13-667-S32.pdf]

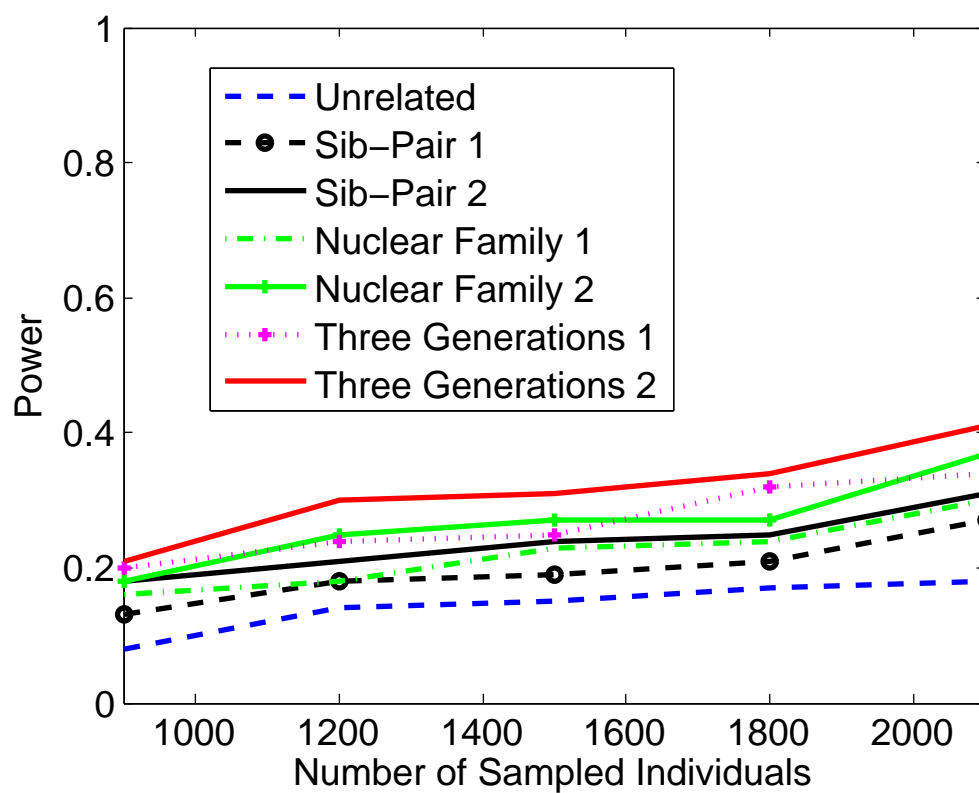

Supplement: Additional file 33: Figure S9A — The power curves of the family-based corrected single marker χ2 statistic under opposite directions of association as a function of the total number of individuals at the significance level α = 0.05 in the test under seven settings: unrelated individuals in cases-controls study, nuclear family groups 1 and 2, sib-pair groups 1 and 2 and three generation family groups 1 and 2, assuming a recessive model, 20% of the risk variants and a baseline penetrance of 0.01. [file 1471-2164-13-667-S33.pdf]

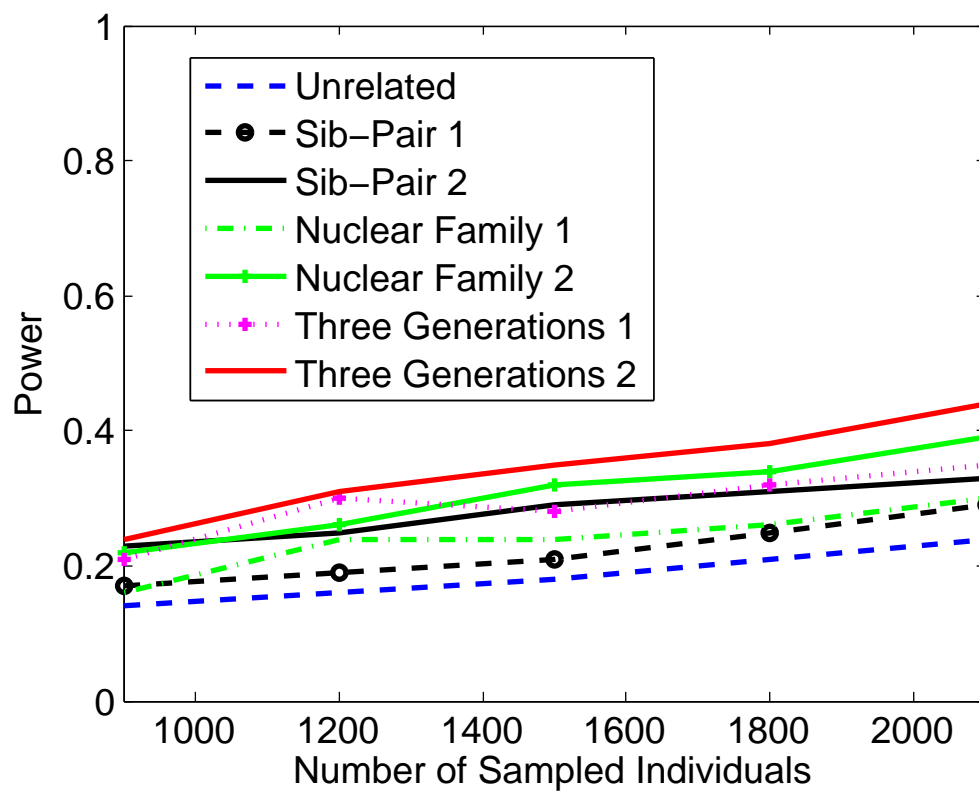

Supplement: Additional file 34: Figure S9B — The power curves of the family-based collapsing test (variants with frequencies ≤0.005 were collapsed) statistic under opposite directions of association as a function of the total number of individuals at the significance level α = 0.05 in the test under seven settings: unrelated individuals in cases-controls study, nuclear family groups 1 and 2, sib-pair groups 1 and 2 and three generation family groups 1 and 2, assuming a recessive model, 20% of the risk variants and a baseline penetrance of 0.01. [file 1471-2164-13-667-S34.pdf]

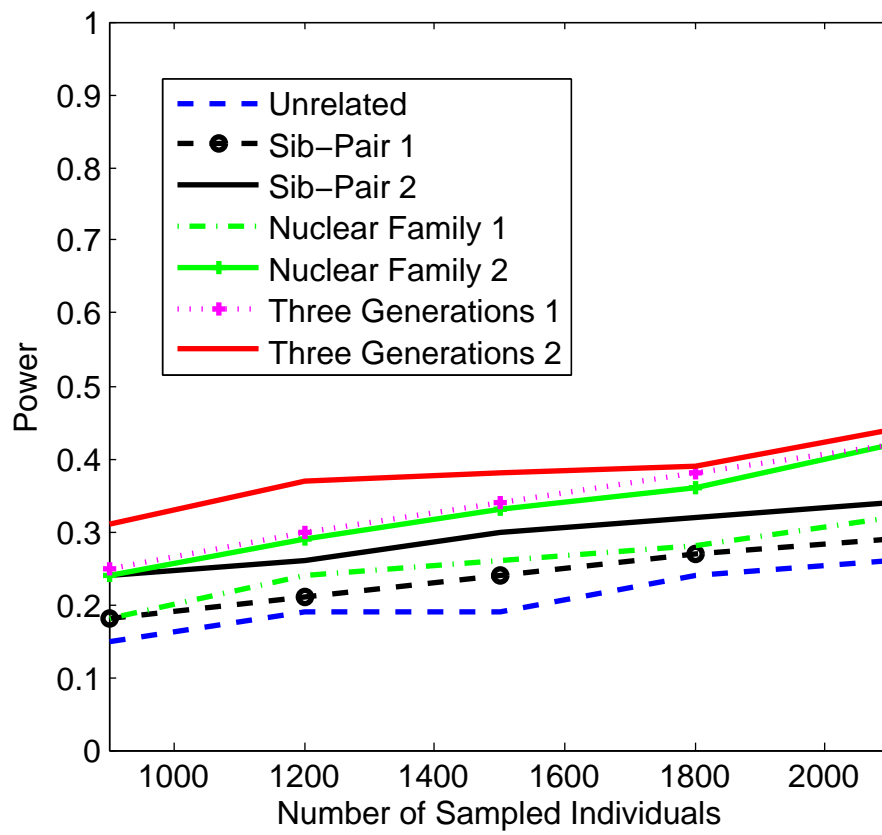

Supplement: Additional file 35: Figure S9C — The power curves of the family-based VT statistic under opposite directions of association as a function of the total number of individuals at the significance level α = 0.05 in the test under seven settings: unrelated individuals in cases-controls study, nuclear family groups 1 and 2, sib-pair groups 1 and 2 and three generation family groups 1 and 2, assuming a recessive model, 20% of the risk variants and a baseline penetrance of 0.01. [file 1471-2164-13-667-S35.pdf]

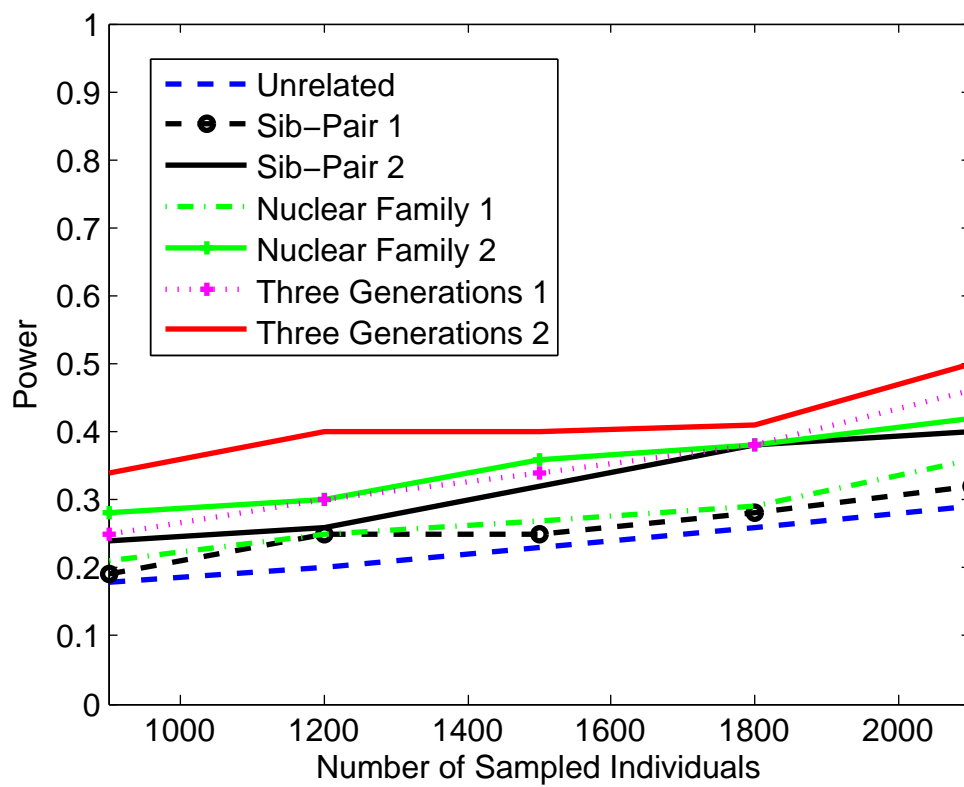

Supplement: Additional file 36: Figure S9D — The power curves of the family-based WSS test statistic under opposite directions of association as a function of the total number of individuals at the significance level α = 0.05 in the test under seven settings: unrelated individuals in cases-controls study, nuclear family groups 1 and 2, sib-pair groups 1 and 2 and three generation family groups 1 and 2, assuming a recessive model, 20% of the risk variants and a baseline penetrance of 0.01. [file 1471-2164-13-667-S36.pdf]
